# The Association between Sulfonylurea Use and All-Cause and Cardiovascular Mortality: A Meta-Analysis with Trial Sequential Analysis of Randomized Clinical Trials

**DOI:** 10.1371/journal.pmed.1001992

**Published:** 2016-04-12

**Authors:** Dimitris Varvaki Rados, Lana Catani Pinto, Luciana Reck Remonti, Cristiane Bauermann Leitão, Jorge Luiz Gross

**Affiliations:** Division of Endocrinology, Hospital de Clínicas de Porto Alegre/Universidade Federal do Rio Grande do Sul, Porto Alegre, Brazil; University of Oxford, UNITED KINGDOM

## Abstract

**Background:**

Sulfonylureas are an effective and inexpensive treatment for type 2 diabetes. There is conflicting data about the safety of these drugs regarding mortality and cardiovascular outcomes. The objective of the present study was to evaluate the safety of the sulfonylureas most frequently used and to use trial sequential analysis (TSA) to analyze whether the available sample was powered enough to support the results.

**Methods and Findings:**

Electronic databases were reviewed from 1946 (Embase) or 1966 (MEDLINE) up to 31 December 2014. Randomized clinical trials (RCTs) of at least 52 wk in duration evaluating second- or third-generation sulfonylureas in the treatment of adults with type 2 diabetes and reporting outcomes of interest were included. Primary outcomes were all-cause and cardiovascular mortality. Additionally, myocardial infarction and stroke events were evaluated. Data were summarized with Peto odds ratios (ORs), and the reliability of the results was evaluated with TSA. Forty-seven RCTs with 37,650 patients and 890 deaths in total were included. Sulfonylureas were not associated with all-cause (OR 1.12 [95% CI 0.96 to 1.30]) or cardiovascular mortality (OR 1.12 [95% CI 0.87 to 1.42]). Sulfonylureas were also not associated with increased risk of myocardial infarction (OR 0.92 [95% CI 0.76 to 1.12]) or stroke (OR 1.16 [95% CI 0.81 to 1.66]). TSA could discard an absolute difference of 0.5% between the treatments, which was considered the minimal clinically significant difference. The major limitation of this review was the inclusion of studies not designed to evaluate safety outcomes.

**Conclusions:**

Sulfonylureas are not associated with increased risk for all-cause mortality, cardiovascular mortality, myocardial infarction, or stroke. Current evidence supports the safety of sulfonylureas; an absolute risk of 0.5% could be firmly discarded.

**Review registration:**

PROSPERO CRD42014004330

## Abstract 2

In a meta-analysis of randomized clinical trials, Dimitris Rados and colleagues find that sulfonylurea use is not associated with an increased risk of all-cause or cardiovascular mortality.

## Editors' Summary

**Background:**

Worldwide, more than 400,000 people have diabetes, a chronic condition characterized by poor glycemic control—dangerously high levels of glucose (sugar) in the blood (hyperglycemia). Blood sugar levels are usually controlled by insulin, a hormone released by beta cells in the pancreas after meals (glucose levels in the blood increase when food is digested and glucose is absorbed). In people with type 2 diabetes (the most common type of diabetes), blood sugar control fails because the fat and muscle cells that normally respond to insulin by removing excess sugar from the blood become resistant to insulin. Type 2 diabetes can often be controlled initially with diet and exercise and with antidiabetic drugs such as metformin (which suppresses glucose production by the liver) and sulfonylureas (which stimulate the secretion of insulin by the pancreas). However, as the disease progresses, the pancreatic beta cells become impaired, and many patients eventually need insulin injections to prevent hyperglycemia. Long-term complications of diabetes, which include an increased risk of cardiovascular problems such as heart attacks (myocardial infarctions) and stroke, reduce the life expectancy of people with diabetes by about ten years compared to people without diabetes.

**Why Was This Study Done?:**

Sulfonylureas have been used for decades to improve glycemic control in people with diabetes, but doubts about their safety were first raised in 1970. Since then, there have been conflicting reports about whether these inexpensive but effective drugs are associated with an increased risk of cardiovascular events and death. Because at least 20%–30% of people with diabetes in high-income countries take second- or third-generation sulfonylureas, such as glipizide and glimepiride, it is important to know whether sulfonylurea use increases the risk of a cardiovascular event or death by even a small amount. Here, the researchers evaluate the safety of the most widely used sulfonylureas by undertaking a systematic review and meta-analysis, with trial sequential analysis, of randomized clinical trials (RCTs) that evaluated second- or third-generation sulfonylureas for the treatment of adults with type 2 diabetes. A systematic review uses predefined criteria to identify all the research on a given topic, a meta-analysis combines the results of several trials, and trial sequential analysis is used to establish whether the sample size of a meta-analysis is sufficiently large to reach firm conclusions about the effect of interventions.

**What Did the Researchers Do and Find?:**

The researchers identified 47 RCTs that met their criteria for inclusion in their study. In total, these RCTs involved 37,650 patients, 890 of whom died during follow-up. Meta-analysis of the results of these trials indicated that sulfonylurea use was not associated with an increased risk of all-cause mortality (death) or cardiovascular mortality. Moreover, sulfonylurea use was not associated with an increased risk of myocardial infarction or stroke. Trial sequential analysis indicated that the sample size in the meta-analysis was large enough that sufficient information was included in the analysis to conclude that fewer than one in 200 patients were likely to have been harmed by the use of sulfonylureas. That is, trial sequential analysis excluded the possibility that—compared to placebo (dummy drug), diet, or an active comparator drug—sulfonylurea use was associated with more than one death (or major cardiovascular event) in every 200 treated patients, which the researchers defined as the minimal clinically significant difference. Importantly, this finding did not change when sulfonylureas were used as an add-on to metformin treatment.

**What Do These Findings Mean?:**

These findings suggest that sulfonylureas are not associated with a clinically significant increased risk for all-cause mortality, cardiovascular mortality, myocardial infarction, or stroke. The accuracy of these findings may be affected by some aspects of the researchers’ analyses, such as the inclusion of studies in their meta-analysis that were not designed to evaluate safety outcomes. Moreover, because this study was not designed to compare different sulfonylureas, further studies are needed to evaluate whether all second- and third-generation sulfonylureas are associated with similar all-cause and cardiovascular mortality risks. Overall, however, these findings are reassuring, and the researchers conclude that sulfonylureas should continue to be used in patients with type 2 diabetes provided their efficacy in controlling hyperglycemia outweighs the risks of weight gain and hypoglycemia (low blood sugar) that are known to be associated with these drugs.

**Additional Information:**

This list of resources contains links that can be accessed when viewing the PDF on a device or via the online version of the article at http://dx.doi.org/10.1371/journal.pmed.1001992.

- The US National Institute of Diabetes and Digestive and Kidney Diseases provides information about diabetes for patients, health-care professionals, and the general public, including information on treatments for diabetes (in English and Spanish)
- The UK National Health Service Choices website provides information for patients and carers about type 2 diabetes, about treatments for type 2 diabetes, and about living with diabetes; it also provides people’s stories about diabetes
- The charity Diabetes UK provides detailed information for patients and carers in several languages, including information on treatments for diabetes
- The UK-based non-profit organization Healthtalkonline has interviews with people about their experiences of diabetes
- MedlinePlus provides links to further resources and advice about diabetes and about medicines for diabetes; it also provides information about metformin and about glipizide, glimepiride, and other sulfonylurea drugs (in English and Spanish)
- More information about this study is available from the PROSPERO International Prospective Register of Systematic Reviews; information about trial sequential analysis is also available

## Introduction

Sulfonylureas are still used frequently in the treatment of patients with type 2 diabetes because they are effective in both improving glycemic control [1] and reducing the microvascular complications of diabetes [2]; in addition, they have the advantage of being inexpensive [3].

There are concerns regarding the safety of sulfonylureas that have persisted from the first randomized clinical trial (RCT) that evaluated sulfonylureas for diabetes treatment (University Group Diabetes Program) [4] until the present time [5–7]. In countries where first-generation sulfonylureas are still in use, they represent only 3% of all oral antihyperglycemic drug prescriptions [8]. Instead, second- and third-generation sulfonylureas are widely used, and it is estimated that 20%–30% of patients with diabetes in developed countries are on sulfonylureas [9,10]. Moreover, a higher proportion (40%–50%) of patients on such treatment have been described in recent multinational cardiovascular studies [11–13].

Observational studies have reported conflicting results regarding sulfonylurea safety [8,14–16], some of them disclosing an association of sulfonylurea use with increased risk of cardiovascular events [8,15]. However, observational studies have limitations because of selection and attrition bias, and from the results one can infer only association, and not causation [17]. There is still a current and intense debate surrounding these safety issues [5,6].

Recent meta-analyses evaluating the safety of sulfonylureas as a group [18–21] or in association with metformin [22] also reported contradictory results. Probably, this was due to the inclusion of observational studies [21,22], the inclusion of first-generation sulfonylureas [19,20], and the lack of evaluation of the risk of type II error [18,20,21]. Analyses that included second- or third-generation sulfonylureas did not report higher risk of mortality or cardiovascular events [18–21].

When dealing with negative results, it is important to evaluate the statistical reliability of the finding, i.e., the power of the analysis. Trial sequential analysis (TSA) is a tool that is increasingly being used [23] to assess whether optimal sample sizes—and benefit or harm boundaries—have been reached by an available sample of patients assuming a minimal clinically significant difference [24]. It has the potential to increase data reliability [24], and its use might be of great benefit in determining whether the currently evaluable evidence about the safety of sulfonylureas is enough to discard falsely positive or negative conclusions [25].

Therefore, the aim of this study was to evaluate the safety of second- and third-generation sulfonylurea use in patients with type 2 diabetes in terms of all-cause and cardiovascular mortality and cardiovascular events (myocardial infarction and stroke), and to quantify the statistical reliability of available data.

## Methods

### Protocol and Registration

We conducted this study using a preconceived protocol according to Cochrane Collaboration recommendations [26] and registered it in the PROSPERO registry (CRD42014004330). This report follows the Preferred Reporting Items for Systematic Reviews and Meta-Analyses (PRISMA) statement [27].

The ethical committee from the research board of Hospital de Clínicas de Porto Alegre exempts systematic reviews from ethical approval.

### Data Sources and Searches

The present study was intended to evaluate the overall safety of the most frequently used sulfonylureas (both second and third generation) in type 2 diabetes through a review of RCTs. Therefore, the search strategy included the terms “type 2 diabetes” and “sulfonylureas” and used the recommended, highly sensitive Cochrane Collaboration strategy for RCT systematic reviews [26]. No outcome or comparator was added to the search terms.

We searched the online databases of MEDLINE (through PubMed), Embase, and the Cochrane Library, as well as conducting a manual review of reference lists of published studies from 1946 (Embase) and 1966 (MEDLINE) up to 31 December 2014. The terms used for searching PubMed are described in S1 Table. We also searched the ClinicalTrials.org registry and the 2014 abstract books of international diabetes meetings (American Diabetes Association and European Association for the Study of Diabetes) for unpublished studies. No time period restrictions were made. All potentially eligible studies were considered for review, limited to the English, Spanish, German, French, Japanese, and Portuguese languages. Three studies were written in languages other than these and were excluded [28–30].

### Study Selection

We included RCTs that evaluated patients with type 2 diabetes who were randomized to receive a second- or third-generation sulfonylurea for at least 52 wk and that reported all-cause or cardiovascular mortality, myocardial infarction, or stroke data. As most of the studies were not specifically designed to evaluate these outcomes, absence of information was frequently observed. In these cases, we attempted to contact the corresponding authors before excluding any study due to lack of data.

We excluded studies comparing sulfonylureas with troglitazone as this medication was withdrawn from the market due to safety issues and is not currently available for clinical use; as rosiglitazone and pioglitazone are still available in some countries, they were included in the analyses. Duplicate reports and extensions of RCTs were also not considered for this review.

### Data Extraction

Two investigators (D. V. R. and L. C. P.) independently evaluated the titles and abstracts of the articles retrieved using the search approach. Abstracts that did not meet the inclusion criteria or that met exclusion criteria were discarded. We selected the remaining studies for full-text evaluation and data extraction. Any disagreements regarding the inclusion or exclusion of a study were solved by consensus, and, if doubt persisted, a third reviewer (C. B. L) evaluated the reference.

We used a standardized form to extract the following details from retrieved studies: first author’s name, publication year and journal, study characteristics (comparator, co-intervention), patient characteristics (mean age, proportion of men/women, and proportion of patients with hypertension, with dyslipidemia, and who were active smokers), study methodology (intervention dosage, frequency, and duration), number of patients included and lost to follow-up, and number of patients with outcomes of interest (all-cause death, cardiovascular death, myocardial infarction, and stroke).

### Quality Assessment

We assessed the included studies in six domains according to the Cochrane Collaboration’s tool for assessing risk of bias [26,31]: (i) random sequence generation, (ii) allocation concealment, (iii) blinding, (iv) incomplete outcome data, (v) selective reporting, and (vi) other bias; for other bias, we evaluated whether the study was conducted with funding support from the pharmaceutical industry. We evaluated the quality of the evidence for each meta-analysis using the Grading of Recommendations Assessment, Development and Evaluation (GRADE) approach. The quality of evidence was classified as “high,” “moderate,” “low,” or “very low.”

Limitations of design or implementation (risk of bias), indirectness of evidence, inexplicable heterogeneity, inconsistent results, and presence of significant publication bias were assessed for each outcome and, if present, decreased the quality ranking of the results for that outcome. The following items were considered to increase the quality of the evidence: a large magnitude of effect, the presence of a dose–response gradient, and if plausible biases worked to decrease the confidence of the finding [32].

### Data Synthesis and Analysis

We compared the outcomes of interest in patients treated with sulfonylureas versus a control group (diet, placebo, or other antihyperglycemic medication). We also performed a meta-analysis separating the controls in classes (diet or placebo and active comparators). We also assessed the use of sulfonylureas as first-line treatment (monotherapy), second-line treatment (in addition to some other medication), or unspecified treatment (when the study did not specify the line of treatment as an inclusion criterion). Because sulfonylureas are commonly used as a second agent in addition to metformin [1,33,34], we also assessed the effects of sulfonylureas when used as an add-on to metformin. We also did exploratory meta-analyses for each sulfonylurea (glibenclamide, glimepiride, glipizide, and gliclazide).

As recommended [26], if a study had more than two intervention groups using different comparators (e.g., rosiglitazone versus metformin versus sulfonylurea), we split the sulfonylurea group sample into two or more groups to avoid falsely increasing the sample size and thereby maintaining the randomization [26].

To evaluate whether the present meta-analysis had sufficient sample size to reach firm conclusions about the effect of interventions [24,25], we performed TSA for the major outcomes. Traditionally, interim analysis of a single trial evaluates whether the monitoring boundaries for a predefined estimated effect are reached before the whole trial population (optimal sample size) has been accrued [24,25]. Similarly, TSA performs a cumulative meta-analysis, which creates a *Z* curve of the summarized observed effect (the cumulative number of included patients and events) and the monitoring boundaries for benefit, harm, and futility, and it estimates the optimal sample size [24,25]. These boundaries and analyses are adjusted to account for the amount of available evidence and to control for repeated analyses, while maintaining type I error at 5% and the power at 80% [24,25]. Therefore, they are initially very wide, but as more information (trials, patients, and events) is included, they become narrower, converging to the unadjusted significance interval. If the *Z* curve of the cumulative meta-analysis crosses one of the boundaries, no further studies are required, and there is sufficient information to support the conclusions. Most importantly, when evaluating treatments that are expected to be not different, the futility boundary allows identification of the “no effect area” as early as possible. As the required number of observations (patients, events) is available, the *Z* curve crosses the futility boundary and identifies that further randomization is not necessary and that it can be affirmed that the intervention does not have the established effect [24,25]. We performed an initial analysis to evaluate the heterogeneity (*I* ^2^)–adjusted optimal sample size for confirming or discarding a harm of an absolute difference between groups of 0.5%, which would lead to a number needed to harm (NNH) of 200 patients.

The current study deals with rare event data and with studies reporting zero events in both arms (double-zero studies). Usual methods (Mantel–Haenszel odds ratio [OR]) used to summarize and aggregate dichotomous variables do not perform as expected in meta-analysis of rare events, and the risk of finding false positives is increased [26,35,36]. Therefore, the studies were summarized using the Peto OR method. This method seems to be better suited to these situations, especially when the incidence of events is near 1% and the effects of intervention are of a small magnitude [36]. As a sensitivity analysis, we performed the analysis with Mantel–Haenszel ORs.

The Peto OR method is not able to use the information from double-zero studies, and these studies are therefore excluded from the analysis. In this setting, it is suggested that a sensitivity analysis with continuity correction be performed [37]. However, TSA software does include double-zero trials in the analysis, using empirical continuity correction. This is performed by adding a constant in the number of events and non-events in both treatment arms. This constant is calculated for each trial and each arm, and this calculation is based on the OR of the meta-analysis (without the double-zero studies to be corrected) and the randomization ratio of the study that needs the empirical continuity correction [25]. Therefore, although our forest plots were constructed using Peto OR analysis (double-zero studies not plotted), double-zero studies were included in the TSA analysis and graphics.

We evaluated the heterogeneity using a Cochran *Q* test, with a threshold *p*-value of 0.1, and an *I* ^2^ test, with a value > 50% indicating high heterogeneity; 95% confidence intervals for *I* ^2^ values were calculated. We also analyzed heterogeneity by using τ^2^ (recommended for small events meta-analyses) [38].

We assessed small study bias by using a contour-enhanced funnel plot, and asymmetry by using Begg and Egger tests. A significant bias was considered if *p* < 0.10. A trim-and-fill computation was used to estimate the effect of missing studies on the interpretation of results.

The main analyses were conducted using Stata version 12.0 (StataCorp) and RevMan version 5.3 (Cochrane Collaboration). The Begg and Egger tests and the trim-and-fill tests were conducted using Stata version 12.0. The empirical continuity correction and TSA were conducted using TSA software version 0.9 (beta) (Copenhagen Trial Unit).

## Results

### Literature Search

**Fig 1:**
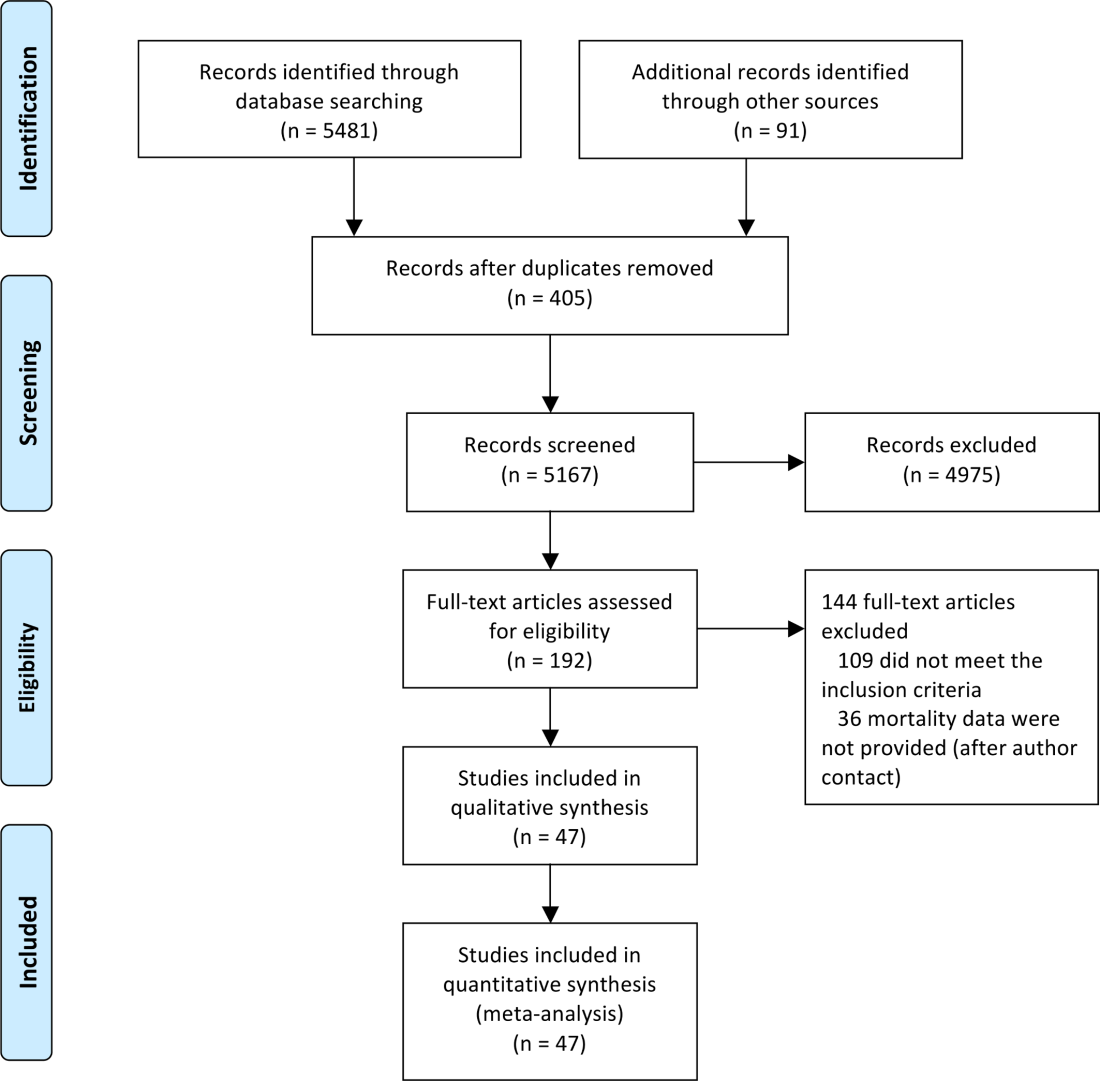
Study flowchart.

We identified 5,572 studies through both the literature and manual searches (Fig 1). After excluding duplicate references and reviewing titles and abstracts, we selected 192 references for full-text evaluation. Of these, 109 trials either did not meet the inclusion criteria or met the exclusion criteria. The main reasons for exclusion were short duration (40 references, 37%), duplicated records (24 references, 22%), and non-randomized study (17 references, 15%). In addition, 36 studies did not report outcome data. Contact information for authors was available from 28 of these studies (19 different authors). Five authors answered our request, but none of them provided additional data. These 36 studies with missing information represented only 10% of the total patient sample. The reviewers had a high agreement rate (κ = 0.917). The final number of studies included was 47 (with 55 pairwise comparisons) [2,39–84], representing 37,650 patients (16,037 randomized to sulfonylureas and 21,613 to comparators). There were 890 all-cause deaths, 354 cardiovascular deaths, 589 myocardial infarctions, and 275 strokes.

### Study Characteristics and Risk of Bias

The included trials were published from 1986 to 2014. The duration of trials varied from 12 to 133 mo. The mean age of the patient population was 57.3 y, and mean baseline HbA_1c_ was 7.2% (minimum 6.8%, maximum 12.2%). Most studies compared sulfonylureas with an active control group. Detailed information about included studies is provided in S2 Table.

We present details regarding the assessment of quality for individual studies and across studies in S1 and S2 Figs. Whether studies used random sequence generation, allocation concealment, and blinding of outcome assessment was unclear in most studies; most studies were considered to be at low risk of bias for the domains blinding of participants and personnel, incomplete outcome data, and selective reporting.

### Sulfonylureas and All-Cause and Cardiovascular Mortality

**Fig 2:**
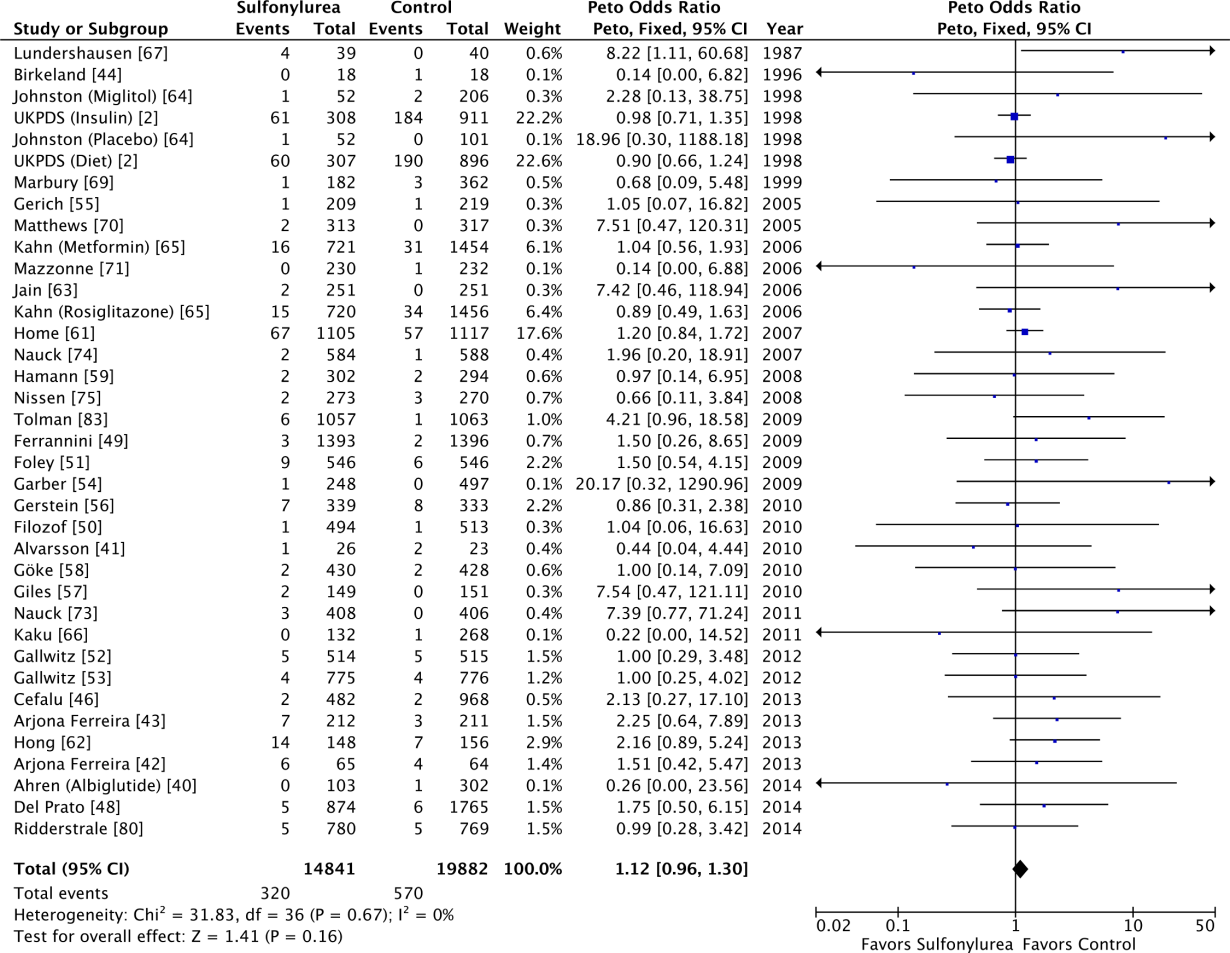
Forest plot for all-cause mortality. For studies with multiple treatment groups, the group being compared is presented in parentheses.

Our meta-analysis did not show a significant association between use of sulfonylureas and all-cause (OR 1.12 [95% CI 0.96 to 1.30]) (Fig 2) or cardiovascular mortality (OR 1.12 [95% CI 0.87 to 1.42]) (S3 Fig). Both analyses have low heterogeneity (all-cause mortality: *I* ^2^ = 0% [95% CI 0% to 17%], *p* for heterogeneity = 0.67; cardiovascular mortality: *I* ^2^ = 12% [95% CI 0% to 20%], *p* for heterogeneity = 0.30). The τ^2^ results were similar to the *I* ^2^ results. The inclusion of double-zero studies with empirical continuity correction analysis did not affect the results (OR 1.11 [95% CI 0.96 to 1.29] and OR 1.12 [95% CI 0.87 to 1.42] for all-cause and cardiovascular mortality, respectively). The sensitivity analyses with Mantel–Haenszel ORs also did not change the results.

We intended to evaluate the long-term safety of sulfonylureas, so to address whether longer studies might show different results, we further restricted the analysis to studies with follow-up of at least 2 y. The results were similar for all-cause (OR 1.05 [95% CI 0.89 to 1.24]) and cardiovascular mortality (OR 1.07 [95% CI 0.83 to 1.39]). We identified small study bias for all-cause mortality. Despite this, the results were unaffected by the trim-and-fill computation: in reality, the point estimation after the computation of theoretical unpublished studies for all-cause mortality was smaller (OR 1.08 [95% CI 0.93 to 1.25]). There was no small study bias for cardiovascular mortality.

### Sulfonylureas and Myocardial Infarction and Stroke

A smaller number of trials reported myocardial infarction and stroke data (23 studies each, comprising 26,521 and 26,175 patients for myocardial infarction and stroke, respectively). We found no significant difference for myocardial infarction in patients treated with sulfonylureas (OR 0.92 [95% CI 0.76 to 1.12]). Including double-zero studies with empirical continuity correction left the results unaffected (OR 0.92 [95% CI 0.76 to 1.12]). In addition, no significant association was observed between sulfonylureas and stroke (OR 1.16 [95% CI 0.81 to 1.66]), and the inclusion of double-zero studies with empirical continuity correction did not change these results (OR 1.16 [95% CI 0.89 to 1.63]). The sensitivity analyses with Mantel–Haenszel ORs also did not change the results for the myocardial infarction and stroke analyses. Small study bias was present for myocardial infarction, but the results were similar with the trim-and-fill computation (OR 0.90 [95% CI 0.74 to 1.09]). No small study bias was identified for stroke events.

### All-Cause and Cardiovascular Mortality with Different Types of Comparators and According to Line of Treatment

**Fig 3:**
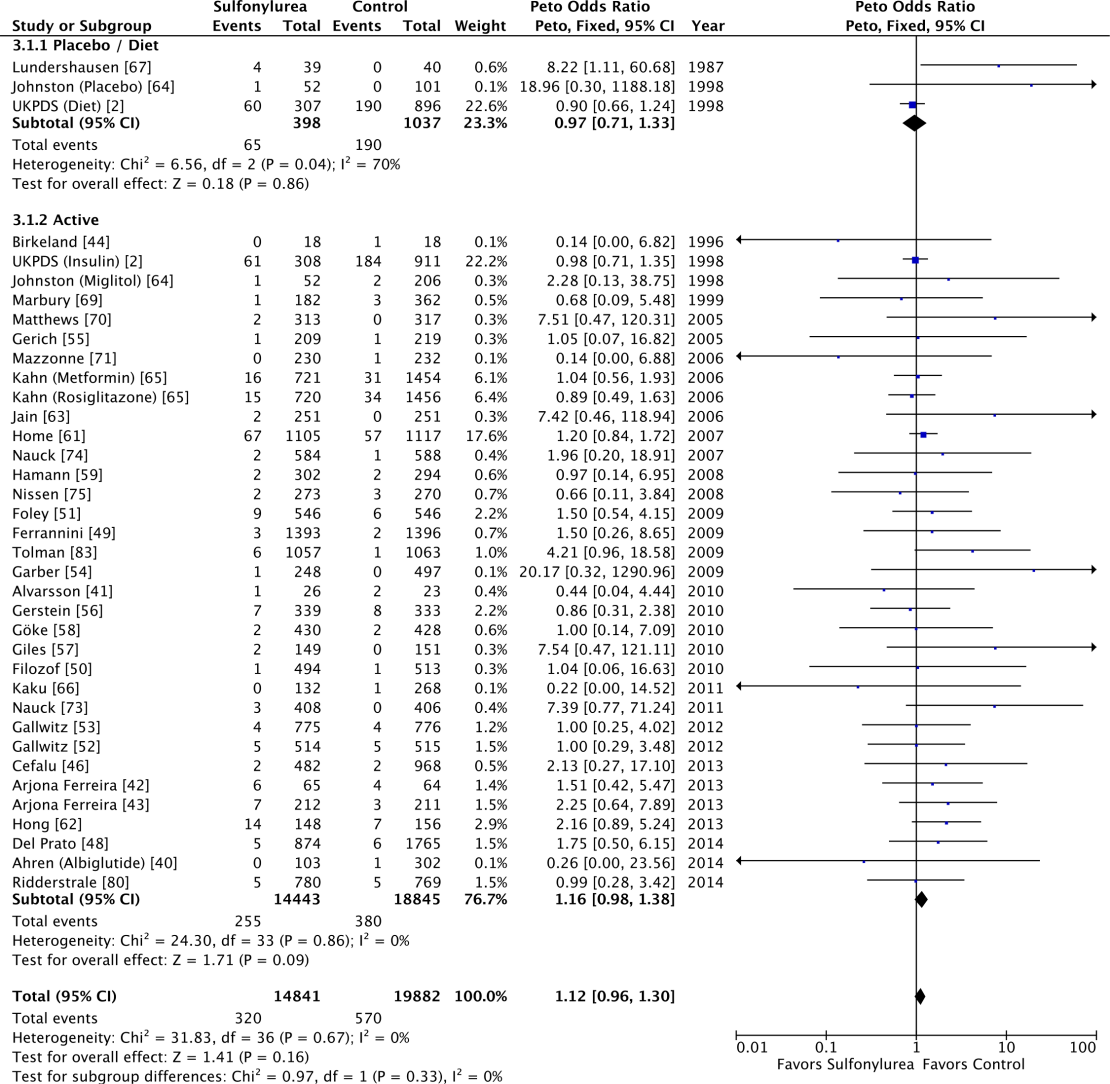
Forest plots for all-cause mortality of sulfonylureas according to comparator (placebo/diet or active comparators). For studies with multiple treatment groups, the group being compared is presented in parentheses.

**Fig 4:**
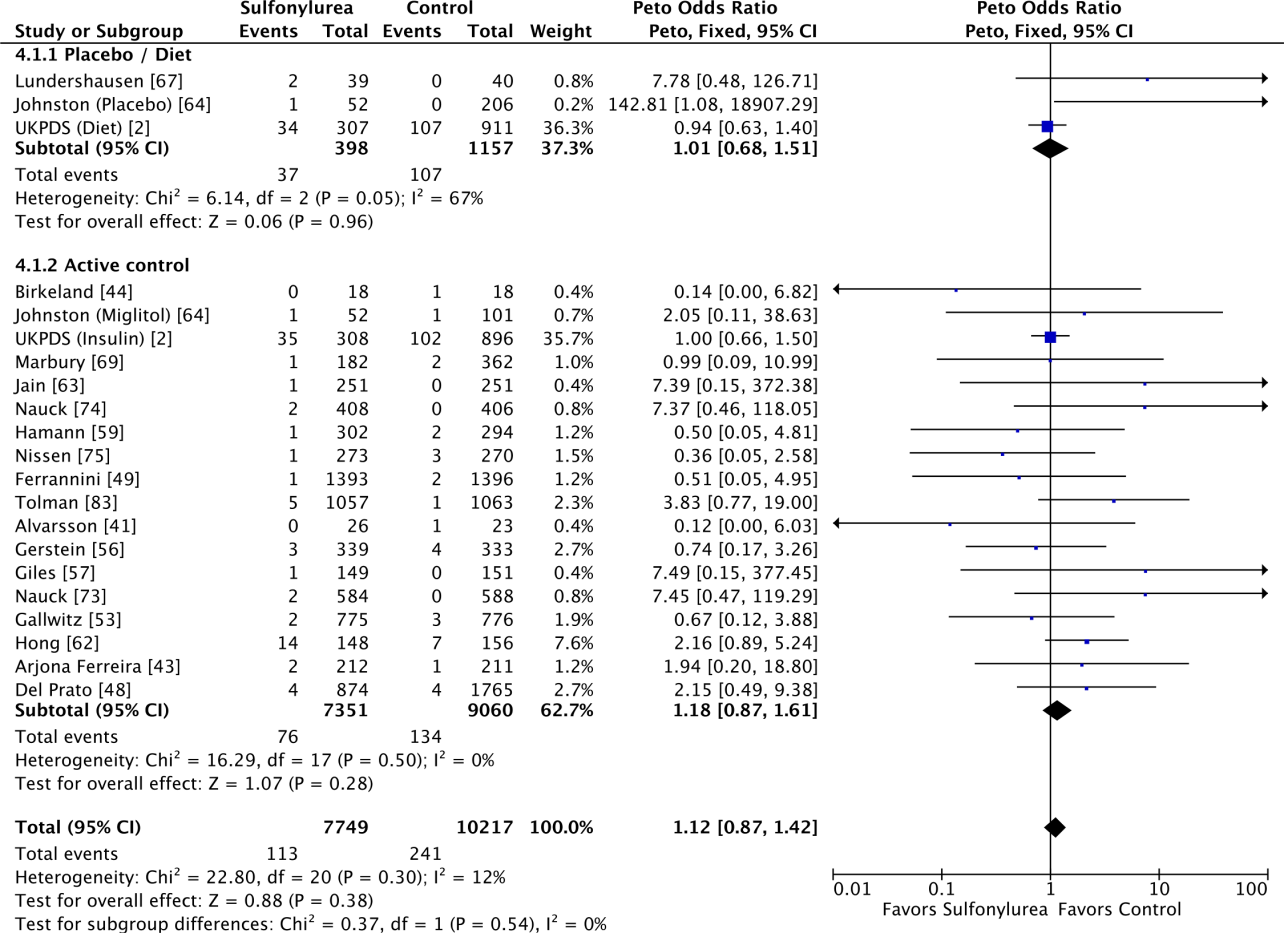
Forest plots for cardiovascular mortality of sulfonylureas according to comparator (placebo/diet or active comparators). For studies with multiple treatment groups, the group being compared is presented in parentheses.

Sulfonylureas were not associated with significant all-cause mortality when compared to placebo or diet (OR 0.97 [95% CI 0.71 to 1.33]; *I* ^2^ = 70% [95% CI 43% to 84%], *p* for heterogeneity = 0.04) or to active comparators (OR 1.16 [95% CI 0.98 to 1.38]; *I* ^2^ = 0% [95% CI 0% to 18%], *p* for heterogeneity = 0.86) (Fig 3). The results for cardiovascular mortality were similar: placebo/diet OR 1.01 (95% CI 0.68 to 1.51; *I* ^2^ = 67% [95% CI 39% to 83%], *p* for heterogeneity = 0.05) and active comparator OR 1.18 (95% CI 0.87 to 1.61; *I* ^2^ = 0% [95% CI 0% to 21%], *p* for heterogeneity = 0.50) (Fig 4). We found also no significant difference in all-cause mortality across all comparator classes individually (S4 Fig).

When stratifying the analysis according to line of treatment, there was no difference in all-cause mortality between treatments irrespective of whether sulfonylureas were used as first-line treatment (OR 1.03 [95% CI 0.86 to 1.24]; *I* ^2^ = 0% [95% CI 0% to 31%], *p* for heterogeneity = 0.50), second-line treatment (OR 1.31 [95% CI 0.98 to 1.74]; *I* ^2^ = 0% [95% CI 0% to 30%], *p* for heterogeneity = 0.88) or treatment line unspecified (OR 1.30 [95% CI 0.63 to 2.67]; *I* ^2^ = 38% [95% CI 0% to 62%], *p* for heterogeneity = 0.17). For cardiovascular mortality, the results were also not affected: first-line treatment OR 1.06 (95% CI 0.81 to 1.39; *I* ^2^ = 14% [95% CI 0% to 40%], *p* for heterogeneity = 0.31), second-line treatment OR 1.42 (95% CI 0.71 to 2.85; *I* ^2^ = 2% [95% CI 0% to 51%], *p* for heterogeneity = 0.41), and treatment line unspecified OR 1.49 (95% CI 0.43 to 5.17; *I* ^2^ = 70% [95% CI 36% to 86%], *p* for heterogeneity = 0.07).

### Sulfonylureas as Add-On to Metformin and All-Cause and Cardiovascular Mortality

**Fig 5:**
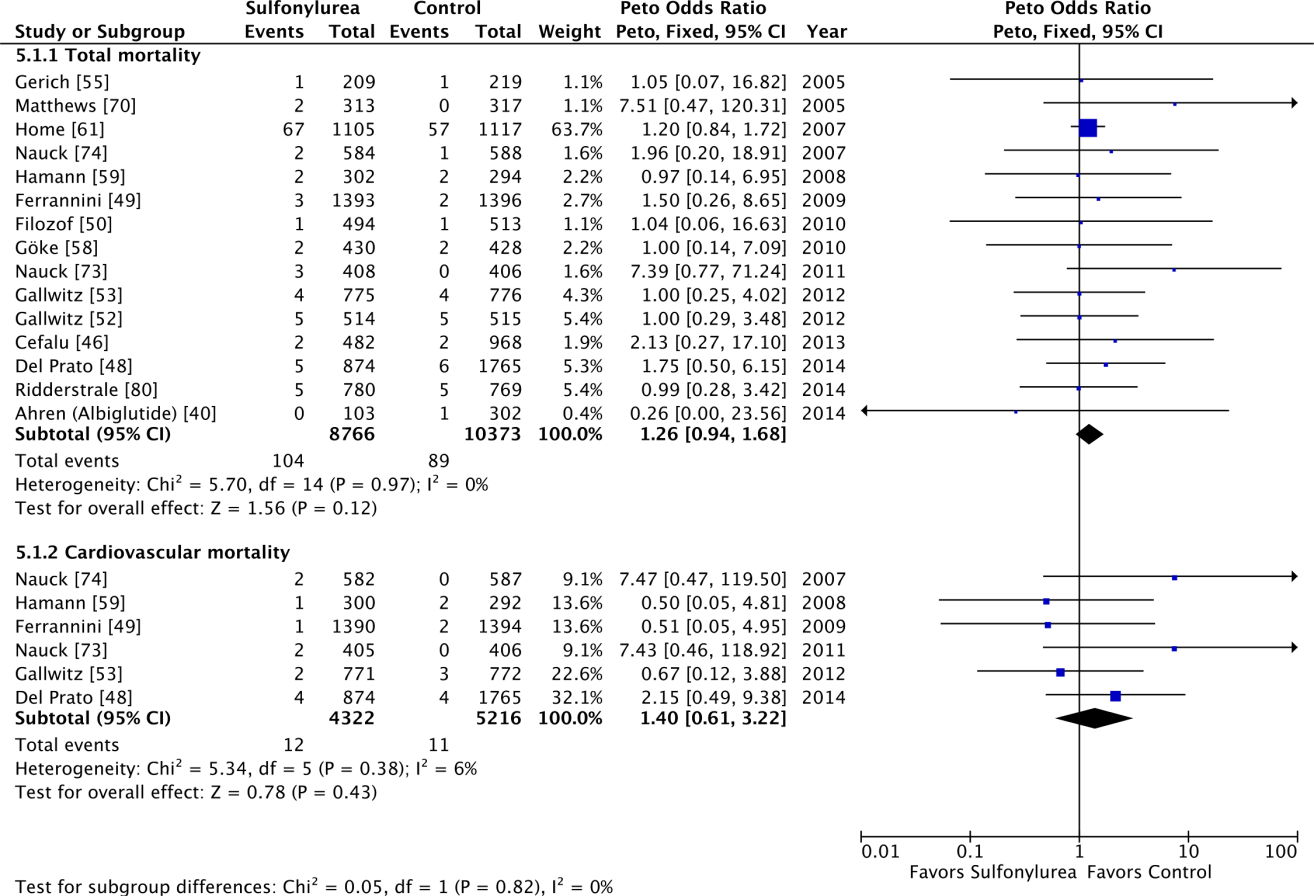
Forest plots for all-cause and cardiovascular mortality of sulfonylureas as an add-on to metformin. For studies with multiple treatment groups, the group being compared is presented in parentheses.

Sulfonylureas as an add-on to metformin were considered safe in terms of overall and cardiovascular mortality (Fig 5), with little heterogeneity: OR 1.26 (95% CI 0.94 to 1.68; *I* ^2^ = 0% [95% CI 0% to 31%], *p* for heterogeneity = 0.97) for all-cause mortality and OR 1.40 (95% CI 0.61 to 3.22; *I* ^2^ = 6% [95% CI 0% to 52%], *p* for heterogeneity = 0.38) for cardiovascular mortality. Including double-zero studies with empirical continuity correction in the analysis did not change these results. All studies in these analyses had active comparators against sulfonylureas.

### Individual Sulfonylurea Agents and Mortality

As an exploratory evaluation, all-cause mortality analysis for each individual sulfonylurea is shown in S5 Fig. Results are similar for cardiovascular mortality. In both analyses, heterogeneity was small. Glipizide was associated with increased all-cause (OR 1.68 [95% CI 1.06 to 2.66]) and cardiovascular mortality (OR 2.1 [95% CI 1.09 to 3.72]), but these analyses are based on a small number of patients and studies.

A sensitivity analysis excluding glipizide trials from the main analyses was performed. We observed a reduction in ORs for all-cause (OR 1.03 [95% CI 0.86 to 1.23]) and cardiovascular mortality (OR 1.00 [95% CI 0.77 to 1.30]). Of note, the futility boundary was still reached in this situation.

### Trial Sequential Analysis

**Fig 6:**
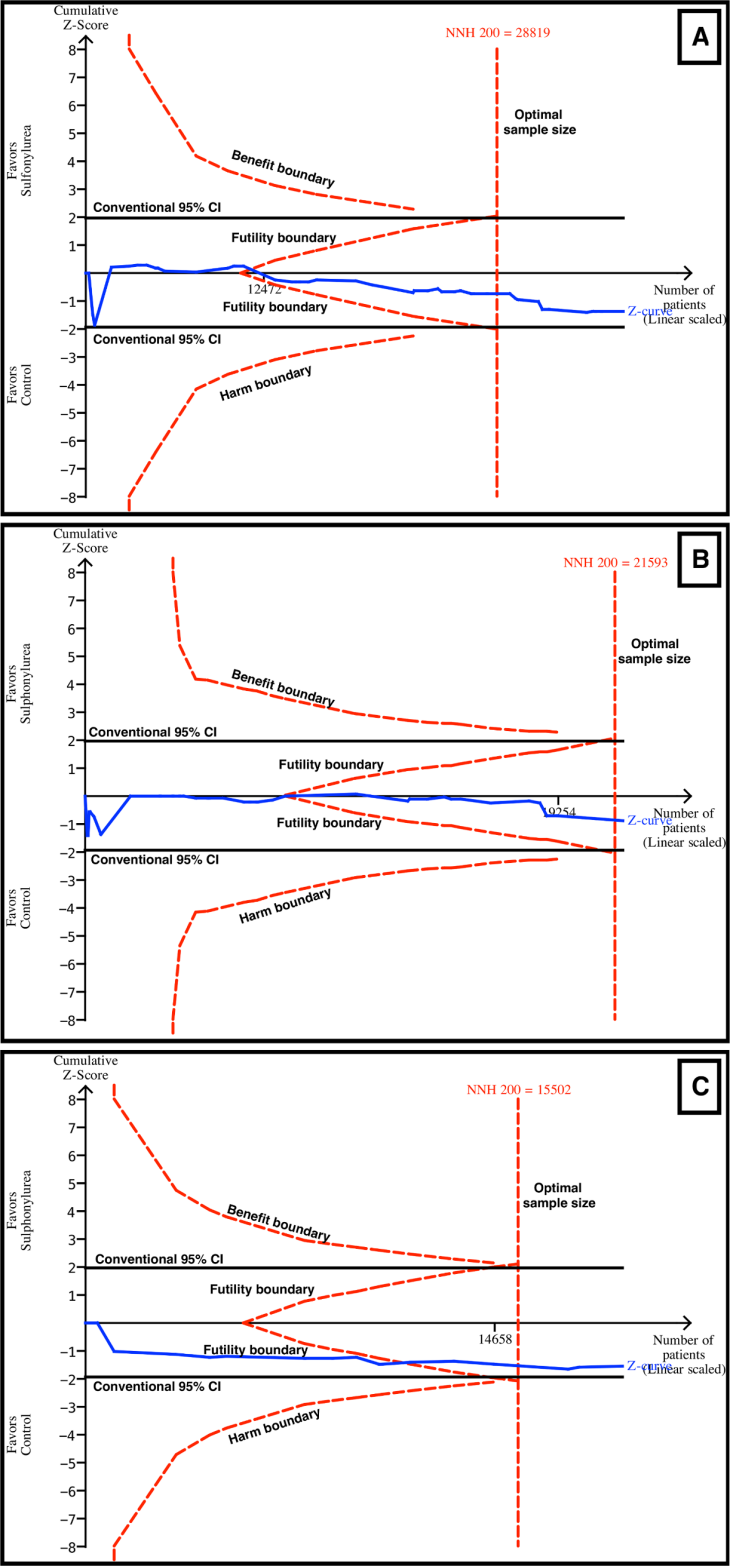
TSA for all-cause and cardiovascular mortality. TSA discarded harm with sulfonylurea use with an α of 5%, a β of 80%, and an absolute difference of 0.5% between the groups (sulfonylurea and comparator). The blue line represents the *Z* curve (cumulative effect size), the red dashed lines represent the harm, benefit, and futility boundaries and the estimated optimal sample size adjusted to sample size and repeated analysis, and the black lines represent the conventional confidence intervals. The black number and marking in the *x*-axis represent the number of patients accrued until that point. (A) Sulfonylureas overall for all-cause mortality. Futility and optimal sample size boundaries were crossed. (B) Sulfonylureas overall for cardiovascular mortality. Futility and optimal sample size boundaries were crossed. (C) Sulfonylureas as add-on to metformin for all-cause mortality. Futility and optimal sample size boundaries were crossed.

TSA evaluates whether there is enough information to reach firm conclusions, and this analysis was performed for the main outcomes in this review. For all-cause and cardiovascular mortality, TSA showed that a NNH of 200 could be discarded, as the number of patients evaluated for all-cause (*n* = 37,650) and cardiovascular mortality (*n* = 21,893) surpassed the optimal sample sizes (*n* = 29,819 for all-cause mortality and *n* = 21,593 for cardiovascular mortality) (Fig 6A and 6B). The combination of sulfonylureas and metformin was evaluated with TSA as well. The *Z* curve surpassed the optimal sample size boundary, and a NNH of 200 could be discarded for all-cause mortality (Fig 6C) but not for cardiovascular mortality. Similarly, for myocardial infarction and stroke, the futility boundaries were reached.

### Meta-Analysis Quality Evaluation and Summary of Findings

The GRADE quality of evidence for all-cause and cardiovascular mortality was high. The identified small study bias does not appear to have skewed the results of the meta-analysis. Financial support from the pharmaceutical industry is a conservative bias, as it might have increased the chance of benefit for the comparator drug [85].

We graded the myocardial infarction and stroke meta-analysis as being of moderate quality, because these outcomes are at greater risk of being skewed due to underreporting and misdiagnosis.

## Discussion

The data presented here suggest that the most frequently used sulfonylureas (second and third generation) are not associated with increased all-cause and cardiovascular mortality in patients with type 2 diabetes. By using TSA, we were able to discard harm at a rate of 1 in every 200 treated patients (i.e., 0.5% of absolute risk) for mortality (all-cause and cardiovascular) and major events (myocardial infarction and stroke). We defined this rate as the minimal clinically significant difference based on a previous study [86]. Furthermore, this finding did not change when sulfonylureas were compared with almost every drug class currently available for the treatment of type 2 diabetes or when sulfonylureas were used as an add-on to metformin.

Other systematic reviews have also evaluated this topic [18–22]. Although some of these studies identified an increased risk of occurrence of mortality or cardiovascular events with sulfonylurea use [19,20,22], others did not find an increased risk [18,21]. These contradictory results may be explained by the inclusion, or not, of first-generation sulfonylureas [19,20], observational studies [21,22], and short-term studies [18–21]. Furthermore, most systematic reviews did not evaluate whether the data presented had enough power to support the conclusions [18,20,21]. We included only RCTs evaluating second- or third-generation sulfonylureas, as monotherapy or in combination. We chose to include only these sulfonylureas, because they are more frequently used than first-generation sulfonylureas [8], alone or in combination with metformin [10].

The current position of regulatory agencies for new drug approval for type 2 diabetes is based on a “safety” approach, with a request that non-inferiority trials be performed to show that a new antihyperglycemic drug has no increased cardiovascular risk compared to placebo [87]. Most recent published large trials have such “no harm” results [11–13]. Although our study might be seen as a non-inferiority safety trial, most of the included studies compared sulfonylureas with active comparators. Therefore, another interpretation of our data is that sulfonylureas are not different from alternative therapies currently available in terms of mortality and cardiovascular outcomes. Furthermore, the US Food and Drug Administration (FDA) states that, for a new drug to treat hyperglycemia to be considered save, the upper limit of the confidence interval of the OR for cardiovascular events must be below 1.30 [87], and the TSA discarded a risk smaller than that. In other words, our study shows that current knowledge can discard a risk small enough to settle the concerns on the safety of sulfonylureas—at least according to the FDA requirements [87].

A particular aspect of our meta-analysis was the use of TSA. This analysis explores the possibility of a false negative result and evaluates the statistical reliability of the present data. To perform this analysis, it is necessary to establish a minimal clinically significant difference in the outcomes between the groups. We chose an absolute difference of 0.5%, which means a NNH of 200. This is half of the risk reported in the Action to Control Cardiovascular Risk in Diabetes (ACCORD) study [86], where an absolute difference of 1% (a NNH of 100) in mortality was found for intensive glucose lowering. We believe using this minimal clinically significant difference for mortality and cardiovascular outcomes is clinically meaningful and provides useful information. This approach allowed us to exclude a risk as small as one death in every 200 treated patients for the evaluated outcomes. Ideally, it would be desirable to be able to exclude a smaller risk, for example a NNH of 500. However, this approach would require a sample of almost 195,000 patients to be randomized. Such a number of individuals will probably never be enrolled, as it is more than five times the number of patients enrolled in sulfonylurea trials in the last 30 years.

Some limitations of the present study must be acknowledged. Unfortunately, we were not able to include all the identified studies in the meta-analyses because the mortality outcomes were not available, even after trying to contact the authors. However, these excluded studies represented only 10% of the study population. It seems unlikely that these data would change the results, as optimal sample size was reached for most analyses. We also could not include three studies because of language restrictions. Some of our analyses were explorative ones (individual sulfonylureas, individual comparators), and the results should be interpreted and used in clinical practice with caution. To assess whether eligible studies were published in the last year, we updated the review of databases (MEDLINE, Embase, and Cochrane Library) with the original strategy up to 9 February 2016. We identified one new study that would fulfill the inclusion criteria of this review [88]. This study included 720 patients randomized to glimepiride or saxagliptin. There was only one death in each group; hence, these additional data did not change our result. Finally, most studies were not designed for assessing long-term safety endpoints, but all of them had a duration of 52 wk or more, which partially controls for this limitation. Although 52 wk may be a short time frame to identify mortality and cardiovascular outcomes, restricting the analysis to longer studies (at least 2 y) did not change the results. Finally, as most of the ORs in our results had a lower limit lower than 1 but close to it, different analysis methods may lead to different results. However, we decreased the uncertainty by performing sensitivity analyses and also explored the consistency of the results by using TSA.

Our study findings are reassuring, as we could discard there being a significantly increased risk with the use of a frequently prescribed antihyperglycemic medication. The sensitivity analyses disclosed that glipizide was associated with an increased risk of mortality; however, it must be stressed that our study was not designed to compare different sulfonylureas, and this result is only exploratory and was based on only a few studies, with a small number of events. A recent network meta-analysis—the preferable approach for comparing drugs that have not been directly compared—showed that second- and third-generation sulfonylureas had similar all-cause and cardiovascular mortality risks [89]. Therefore, this finding regarding glipizide must be further evaluated in future studies. Whether second- and third-generation sulfonylureas should be considered as a class or as individual agents has yet to be determined.

Another important unresolved question is which drug should be added to patients who are failing metformin monotherapy. To date, no antihyperglycemic agent used in association with metformin has reduced mortality or cardiovascular events. Even the recent published trials of dipeptidyl peptidase-4 inhibitors in patients with type 2 diabetes and high cardiovascular risk did not show a reduction in cardiovascular events [11–13], but there was a concern regarding heart failure incidence in two of the trials [12,90]. Our data show that second- and third-generation sulfonylureas are a safe option, but we hope that newer drugs will do better than that and will be able to decrease cardiovascular events and mortality risks compared to sulfonylureas. Although the EMPA-REG study did not directly explore this issue, the results suggest that empagliflozin might be such a drug, as it was able to reduce the risk of cardiovascular events and mortality (all cause and cardiovascular) [91]. To clarify the question of which should be the preferred drug for patients failing metformin, the Cardiovascular Outcome Study of Linagliptin versus Glimepiride in Patients with Type 2 Diabetes (CAROLINA) and the Glycemia Reduction Approaches in Diabetes: A Comparative Effectiveness Study (GRADE) results are awaited [92,93], as they will further evaluate sulfonylureas against newer drug classes in the long term.

In conclusion, the present study suggests that the use of second- and third-generation sulfonylureas in patients with type 2 diabetes is not associated with increased cardiovascular risk and all-cause mortality, irrespective of comparator or background medication. Sulfonylureas should therefore still be used; however, it is important to weigh their efficacy in controlling hyperglycemia and low cost against the risks of hypoglycemia and weight gain.

## Supporting Information

S1 Fig Quality assessment across studies. (PDF)

S2 Fig Quality assessment for individual studies. (PDF)

S3 Fig Forest plot for cardiovascular mortality. (PDF)

S4 Fig Forest plot for all-cause mortality across individual comparators. (PDF)

S5 Fig Forest plot for all-cause mortality for different sulfonylureas. (PDF)

S1 Table Search strategy for PubMed. (DOCX)

S2 Table Included randomized clinical trials and their baseline characteristics. (DOCX)

S1 Text Protocol of the review. (PDF)

S2 Text Checklist of PRISMA Statement. (DOC)

## Abbreviations

NNH: number needed to harm
OR: odds ratio
RCT: randomized clinical trial
TSA: trial sequential analysis
