# Correction: The Association between Sulfonylurea Use and All-Cause and Cardiovascular Mortality: A Meta-Analysis with Trial Sequential Analysis of Randomized Clinical Trials

**DOI:** 10.1371/journal.pmed.1002091

**Published:** 2016-06-24

**Authors:** Dimitris Varvaki Rados, Lana Catani Pinto, Luciana Reck Remonti, Cristiane Bauermann Leitão, Jorge Luiz Gross

## Notice of Republication

This article was republished on June 14^th^, 2016, to correct errors in the article citation. The following authors’ names were indexed incorrectly: Dimitris Varvaki Rados, Lana Catani Pinto, Luciana Reck Remonti, Cristiane Bauermann Leitão. The updated citation is available here. Please download this article again to view the correct version.
