# Supplementary figures and images for: The Association between Sulfonylurea Use and All-Cause and Cardiovascular Mortality: A Meta-Analysis with Trial Sequential Analysis of Randomized Clinical Trials
Source: PLoS Med. 2016 Apr 12;13(4):e1001992. doi: 10.1371/journal.pmed.1001992 (PMC4829174; doi:10.1371/journal.pmed.1001992)

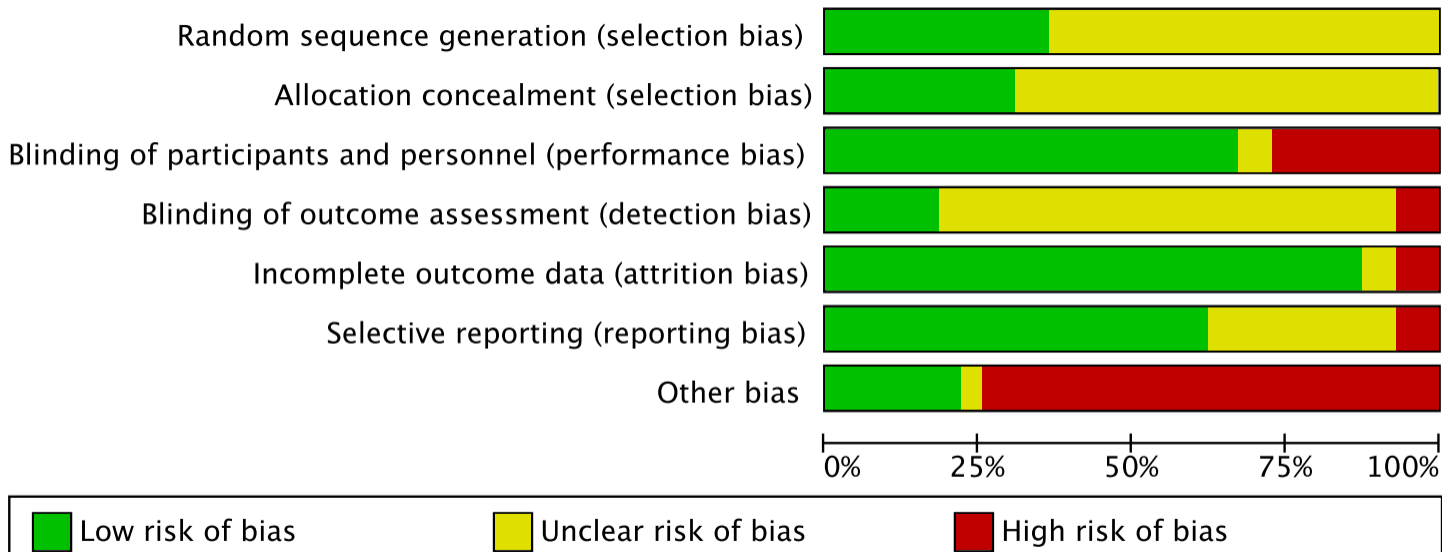

Supplement: S1 Fig — (PDF) [file pmed.1001992.s001.pdf]

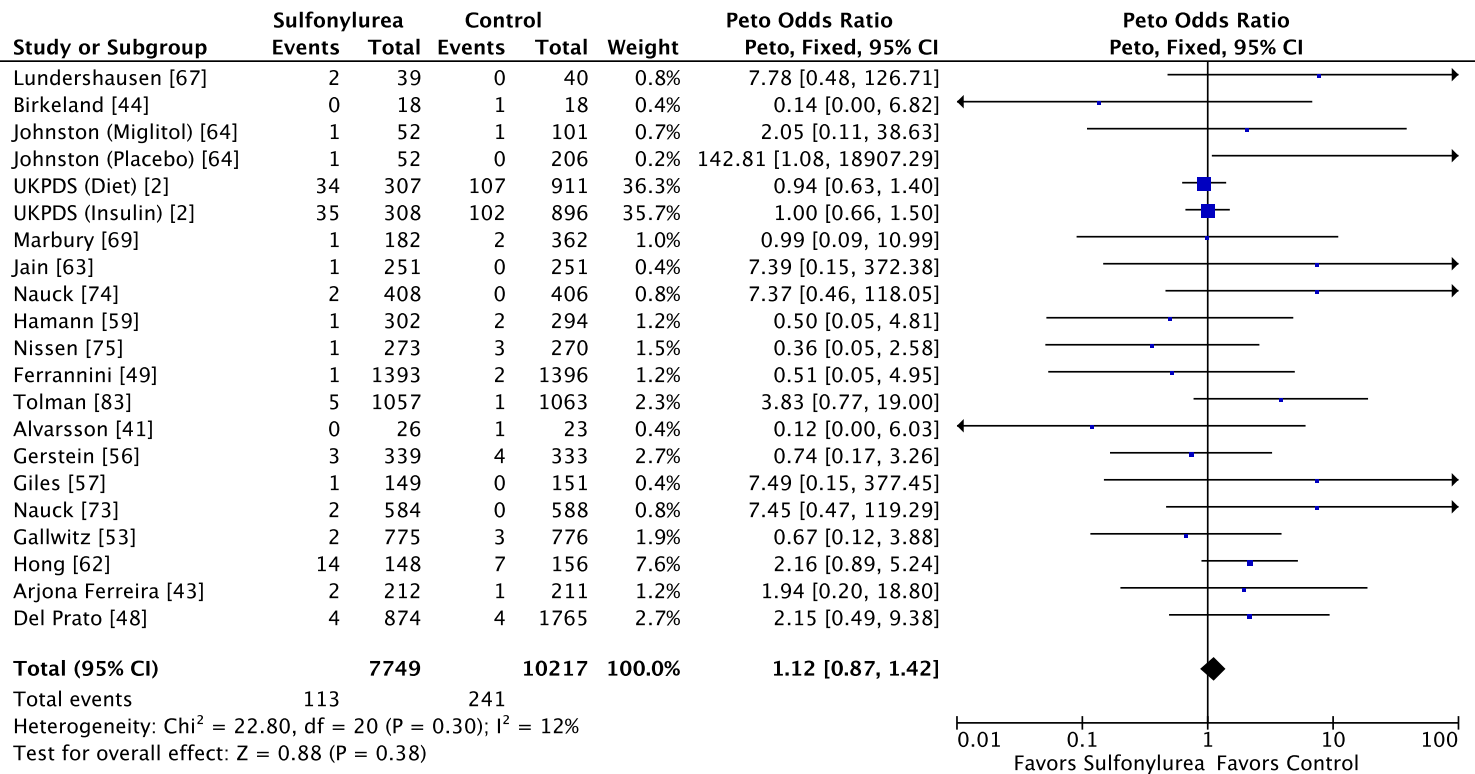

Supplement: S3 Fig — (PDF) [file pmed.1001992.s003.pdf]

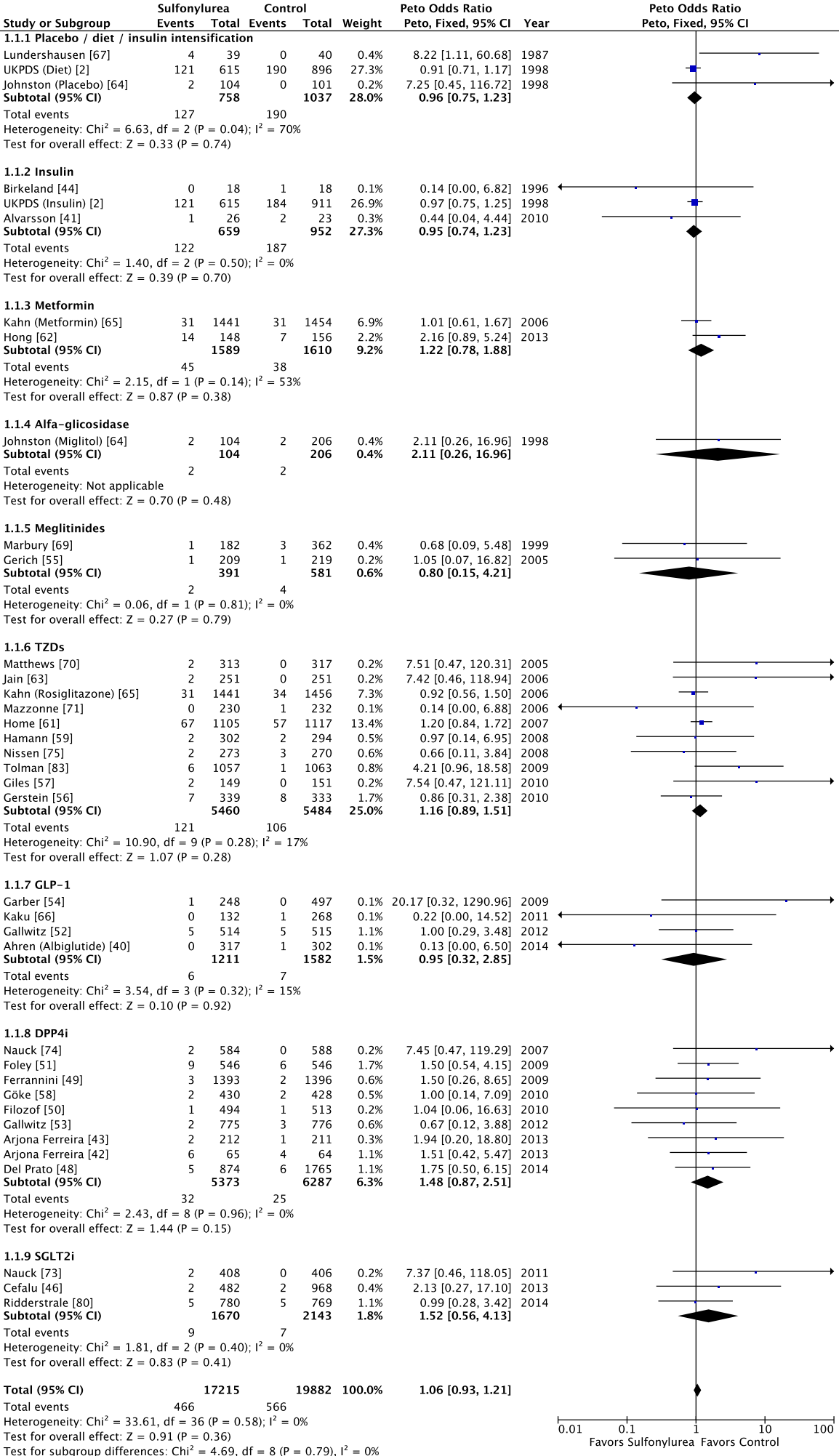

Supplement: S4 Fig — (PDF) [file pmed.1001992.s004.pdf]

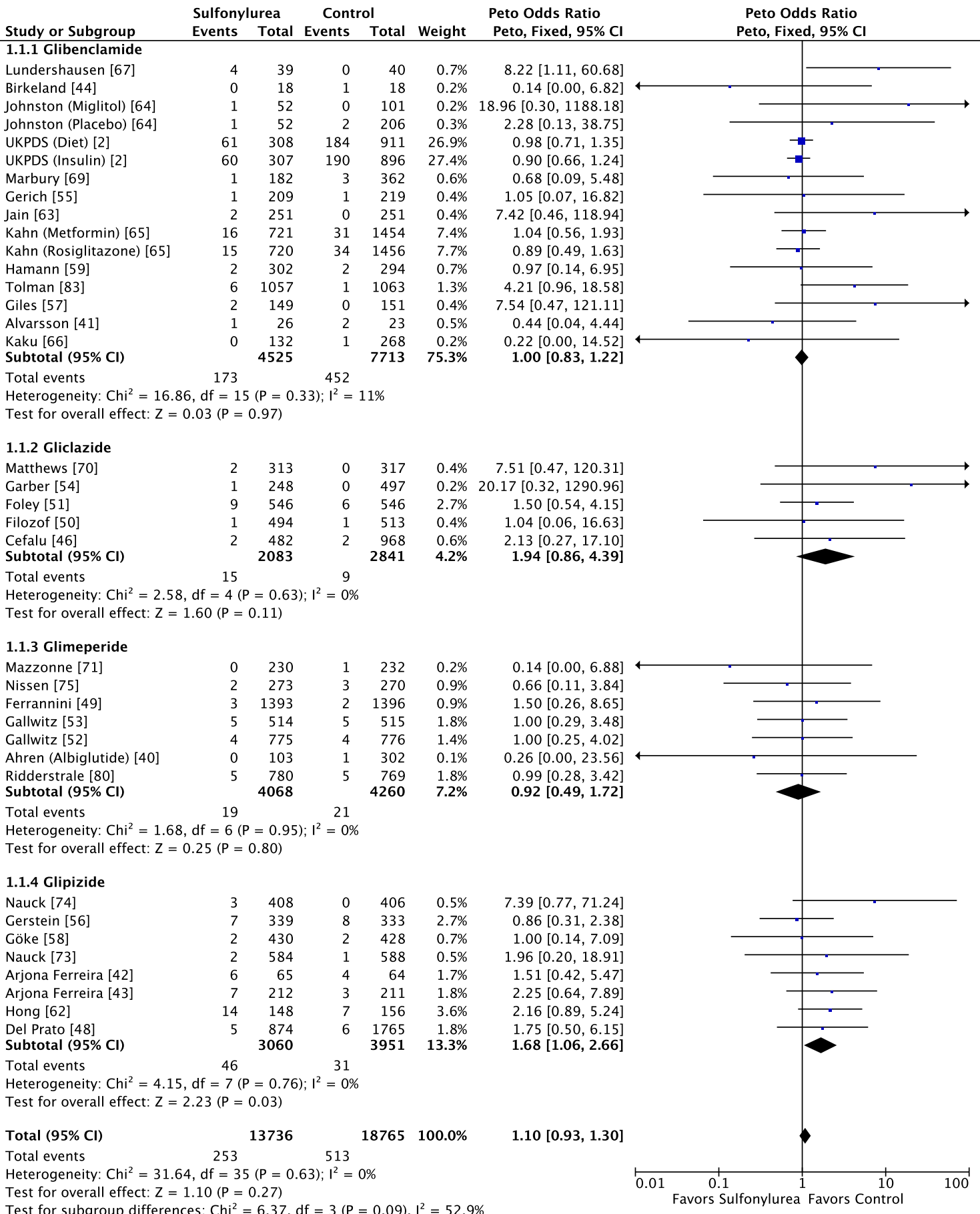

Supplement: S5 Fig — (PDF) [file pmed.1001992.s005.pdf]
