# Supplementary material for: The Association between Sulfonylurea Use and All-Cause and Cardiovascular Mortality: A Meta-Analysis with Trial Sequential Analysis of Randomized Clinical Trials
Source: PLoS Med. 2016 Apr 12;13(4):e1001992. doi: 10.1371/journal.pmed.1001992 (PMC4829174; doi:10.1371/journal.pmed.1001992)
Supplement: S2 Fig — (PDF) [file pmed.1001992.s002.pdf]

	Random sequence generation (selection bias)	Allocation concealment (selection bias)	Blinding of participants and personnel (performance bias)	Blinding of outcome assessment (detection bias)	Incomplete outcome data (attrition bias)	Selective reporting (reporting bias)	Other bias
Abbatecola [39]	?	?	−	?	−	+	+
Ahren (Albiglutide) [40]	?	?	+	?	+	?	−
Ahrén (Placebo) [40]	?	?	+	?	+	?	−
Ahrén (Sitagliptin) [40]	?	?	+	?	+	?	−
Alvarsson [41]	?	?	−	−	+	+	+
Arjona Ferreira [42]	+	+	+	?	+	+	−
Arjona Ferreira [43]	+	+	+	?	+	+	−
Birkeland [44]	?	?	−	?	+	−	−
Campbell [45]	?	?	−	?	+	?	?
Cefalu [46]	+	+	+	+	+	+	−
Clauson [47]	?	?	−	?	+	−	?
Del Prato [48]	?	?	+	+	+	+	−
Ferrannini [49]	?	?	+	?	+	+	−
Filozof [50]	?	?	+	?	+	+	−
Foley [51]	?	?	+	?	+	+	−
Gallwitz [52]	+	+	−	−	−	?	−
Gallwitz [53]	+	+	+	+	−	+	−
Garber [54]	+	+	+	−	−	+	−
Gerich [55]	?	?	+	?	+	?	−
Gerstein [56]	?	?	+	+	+	+	−
Giles [57]	?	?	+	?	+	+	−
Göke [58]	+	+	+	?	+	+	−
Hamann [59]	+	+	+	?	+	+	−
Hanefeld [60]	?	?	+	?	+	+	−
Home [61]	+	+	−	+	+	+	−
Hong [62]	+	+	+	?	+	+	+
Jain [63]	?	?	+	?	+	+	−
Johnston (Miglitol) [64]	?	?	+	?	?	?	−
Johnston (Placebo) [64]	?	?	+	?	?	?	−
Kahn (Metformin) [65]	+	+	+	+	+	+	−
Kahn (Rosiglitazone) [65]	+	+	+	+	+	+	−
Kaku [66]	?	?	−	−	+	−	−
Lundershausen [67]	?	?	+	?	+	−	+
Madsbad [68]	?	?	+	?	+	?	−
Marbury [69]	?	?	+	?	+	+	−
Matthews [70]	?	?	+	?	?	?	−
Mazzonne [71]	?	?	+	+	+	+	−
Nakamura (Voglibose) [72]	+	?	?	?	+	?	+
Nakmura (Natglinide) [72]	+	?	?	?	+	?	+
Nakmura (Piogltazne) [72]	+	?	?	?	+	?	+
Nauck [73]	+	+	+	?	+	+	−
Nauck [74]	?	?	+	?	+	+	−
Nissen [75]	+	+	+	+	+	+	−
Perriello [76]	?	?	+	?	+	+	−
Petrica [77]	?	?	−	+	+	+	+
Petrica [78]	?	?	−	?	+	?	+
Quatraro [79]	?	?	−	?	+	?	+
Ridderstrale [80]	?	?	+	?	+	+	−
Ristic [81]	?	?	+	?	+	+	−
Rosenstock [82]	?	?	+	?	+	+	−
Tolman [83]	+	+	+	?	+	+	−
UKPDS (Diet) [2]	+	+	−	?	+	+	−
UKPDS (Insulin) [2]	+	+	−	?	+	+	−
Vähätalo (Insulin) [84]	?	?	−	?	+	?	+
Vähätalo (Metformin) [84]	?	?	−	?	+	?	+
