# Supplementary material for: The Association between Sulfonylurea Use and All-Cause and Cardiovascular Mortality: A Meta-Analysis with Trial Sequential Analysis of Randomized Clinical Trials
Source: PLoS Med. 2016 Apr 12;13(4):e1001992. doi: 10.1371/journal.pmed.1001992 (PMC4829174; doi:10.1371/journal.pmed.1001992)
Supplement: S1 Table — (DOCX) [file pmed.1001992.s006.docx]

| ("Glyburide"[Mesh]) OR ("glibornuride" [Supplementary Concept]) OR ("Glipizide"[Mesh]) OR ("gliquidone" [Supplementary Concept]) OR ("glisoxepide" [Supplementary Concept]) OR ("glyclopyramide" [Supplementary Concept]) OR ("glimepiride" [Supplementary Concept]) OR ("Gliclazide"[Mesh]) AND ("Diabetes Mellitus, Type 2"[Mesh]) AND (randomized controlled trial[pt] OR controlled clinical trial[pt] OR randomized controlled trials[mh] OR random allocation[mh] OR double-blind method[mh] OR single-blind method[mh] OR clinical trial[pt] OR clinical trials[mh] OR (“clinical trial”[tw]) OR ((singl*[tw] OR doubl*[tw] OR trebl*[tw] OR tripl*[tw]) AND (mask*[tw] OR blind*[tw])) OR (“latin square”[tw]) OR placebos[mh] OR placebo*[tw] OR random*[tw] OR research design[mh: noexp] OR follow-up studies[mh] OR prospective studies[mh] OR cross-over studies[mh] OR control*[tw] OR prospectiv*[tw] OR volunteer*[tw]) NOT (animal[mh] NOT human[mh]) |
| --- |
