# Supplementary material for: The Association between Sulfonylurea Use and All-Cause and Cardiovascular Mortality: A Meta-Analysis with Trial Sequential Analysis of Randomized Clinical Trials
Source: PLoS Med. 2016 Apr 12;13(4):e1001992. doi: 10.1371/journal.pmed.1001992 (PMC4829174; doi:10.1371/journal.pmed.1001992)
Supplement: S2 Table — (DOCX) [file pmed.1001992.s007.docx]

| Author | Year | Interventions | Number of patients | Mean age | Baseline HbA1c % | Follow-up (months) |
| --- | --- | --- | --- | --- | --- | --- |
| Abbatecola[39] | 2006 | Glibenclamide | 79 | 74.3 | 7.2 | 12 |
|  |  | Repaglinide | 77 | 74.5 | 7.3 |  |
| Ahrén[40] | 2014 | Glimeperide | 307 | 54.5 | N.R. | 36 |
|  |  | Sitagliptin | 302 | 54.5 | N.R. |  |
|  |  | Albiglutide | 302 | 54.5 | N.R. |  |
|  |  | Placebo | 101 | 54.5 | N.R. |  |
| Alvarsson[41] | 2010 | Glibenclamide | 26 | 55.9 | 6.8 | 72 |
|  |  | Insulin | 23 | 51.7 | 7.1 |  |
| Arjona Ferreira[42] | 2013 | Glimeperide | 65 | 60 | 7.8 | 12 |
|  |  | Sitagliptin | 64 | 60 | 7.9 |  |
| Arjona Ferreira[43] | 2013 | Glipizide | 212 | 64.2 | 7.8 | 12 |
|  |  | Sitagliptin | 211 | 64.2 | 7.8 |  |
| Bikeland[44] | 1996 | Glibenclamide | 18 | 59.2 | 8.5 | 42 |
|  |  | Insulin | 18 | 59.2 | 9.1 |  |
| Campbell[45] | 1994 | Glipizide | 24 | 57 | 11.8 | 12 |
|  |  | Metformin | 24 | 57 | 11.5 |  |
| Cefalu[46] | 2013 | Gliclazide | 482 | 56.2 | 7.8 | 12 |
|  |  | Canagliflozin | 968 | 56.2 | 7.8 |  |
| Clauson[47] | 1996 | Glibenclamide | 20 | 59.3 | 10.3 | 12 |
|  |  | Nothing (both arms on insulin) | 19 | 57.8 | 9.8 |  |
| Delprato[48] | 2014 | Glipizide | 874 | 55.4 | 7.6 | 24 |
|  |  | Alogliptin | 1765 | 55.4 | 7.6 |  |
| Ferranini[49] | 2009 | Glimeperide | 1393 | 57.5 | 7.3 | 12 |
|  |  | Vildagliptin | 1396 | 57.5 | 7.3 |  |
| Filozof[50] | 2010 | Gliclazide | 494 | 59.5 | 8.5 | 12 |
|  |  | Vildagliptin | 513 | 59.5 | 8.5 |  |
| Foley[51] | 2009 | Gliclazide | 546 | 54.3 | 8.7 | 12 |
|  |  | Vildagliptin | 546 | 55.2 | 8.6 |  |
| Gallwitz[53] | 2012 | Glimeperide | 775 | 59.8 | 7.7 | 24 |
|  |  | Linagliptin | 776 | 59.8 | 7.7 |  |
| Gallwitz[52] | 2012 | Glimeperide | 514 | 60 | 7.4 | 24 |
|  |  | Exenatide | 515 | 60 | 7.4 |  |
| Garber[54] | 2009 | Gliclazide | 248 | 53 | 8.3 | 48 |
|  |  | Liraglutide | 497 | 53 | 8.3 |  |
| Gerich[55] | 2005 | Glibenclamide | 209 | 52.6 | 8.3 | 24 |
|  |  | Nateglinide | 219 | 53.5 | 8.4 |  |
| Gerstein[56] | 2010 | Glipizide | 339 | 61 | 7.2 | 18 |
|  |  | Rosiglitazone | 333 | 61 | 7.1 |  |
| Giles[57] | 2010 | Glibenclamide | 149 | 64 | 8.3 | 12 |
|  |  | Pioglitazone | 151 | 64 | 8.6 |  |
| Göke[58] | 2010 | Glipizide | 430 | 57.6 | 7.7 | 12 |
|  |  | Saxagliptin | 428 | 57.6 | 7.7 |  |
| Hamann[59] | 2008 | Glibenclamide | 302 | 60 | 8.0 | 12 |
|  |  | Rosiglitazone | 294 | 60 | 8.0 |  |
| Hanefeld[60] | 2007 | Glibenclamide | 203 | 60.4 | 8.2 | 12 |
|  |  | Rosiglitazone | 384 | 60.4 | 8.2 |  |
| Home[61] | 2007 | Any 2nd / 3rd generation sulphonylurea | 1105 | 57 | N.R | 45 |
|  |  | Rosiglitazone | 1117 | 57 | N.R |  |
| Hong[62] | 2013 | Glipizide | 148 | 63.3 | 7.6 | 36 |
|  |  | Metformin | 156 | 63.3 | 7.6 |  |
| Jain[63] | 2006 | Glibenclamide | 251 | 52.1 | 9.2 | 13 |
|  |  | Pioglitazone | 251 | 52.1 | 9.2 |  |
| Johnston[64] | 1998 | Glibenclamide | 104 | 67.7 | 8.4 | 12 |
|  |  | Miglitol | 206 | 67.4 | 8.4 |  |
|  |  | Placebo | 101 | 68.5 | 8.3 |  |
| Kahn[65] | 2006 | Glibenclamide | 1441 | 56.4 | 7.3 | 48 |
|  |  | Metformin | 1454 | 57.9 | 7.3 |  |
|  |  | Rosiglitazone | 1456 | 56.3 | 7.3 |  |
| Kaku[66] | 2011 | Glibenclamide | 132 | 58.3 | 9.2 | 12 |
|  |  | Liraglutide | 268 | 58.3 | 9.3 |  |
| Lunderhausen[67] | 1987 | Glibenclamide | 39 | 61 | N.R. | 12 |
|  |  | Placebo | 40 | 61 | N.R. |  |
| Madsbad[68] | 2001 | Glipizide | 81 | 62 | 7.2 | 12 |
|  |  | Repaglinide | 175 | 60.2 | 7.3 |  |
| Marbury[69] | 1999 | Glibenclamide | 182 | 58 | 9.0 | 12 |
|  |  | Repaglinide | 362 | 58 | 8.7 |  |
| Matthews[70] | 2005 | Gliclazide | 313 | 56 | 8.5 | 12 |
|  |  | Pioglitazone | 317 | 57 | 8.7 |  |
| Mazzonne[71] | 2006 | Glimeperide | 230 | 59 | 7.4 | 18 |
|  |  | Pioglitazone | 232 | 59 | 7.4 |  |
| Nakamura[72] | 2006 | Glibenclamide | 21 | 53 | 7.8 | 12 |
|  |  | Voglibose | 17 | 55 | 7.6 |  |
|  |  | Pioglitazone | 17 | 56 | 8.0 |  |
|  |  | Nateglinide | 16 | 53 | 7.7 |  |
| Nauck[74] | 2007 | Glipizide | 584 | 56 | 7.5 | 12 |
|  |  | Sitagliptin | 588 | 56 | 7.5 |  |
| Nauck[73] | 2011 | Glipizide | 408 | 58.4 | 7.7 | 12 |
|  |  | Dapagliflozin | 406 | 58.4 | 7.7 |  |
| Nissen[75] | 2008 | Glimeperide | 273 | 59 | 7.4 | 18 |
|  |  | Pioglitazone | 270 | 59 | 7.4 |  |
| Perriello[76] | 2007 | Gliclazide | 135 | 59 | 8.7 | 12 |
|  |  | Pioglitazone | 140 | 58 | 8.7 |  |
| Petrica[77] | 2009 | Glimeperide | 17 | 63 | 7.6 | 12 |
|  |  | Rosiglitazone | 17 | 63 | 7.7 |  |
| Petrica[78] | 2011 | Glimeperide | 39 | 58 | 7.5 | 12 |
|  |  | Pioglitazone | 39 | 56 | 7.7 |  |
| Quatraro[79] | 1986 | Gliclazide | 15 | 56 | 12.2 | 12 |
|  |  | Nothing (both arms on insulin) | 15 | 57 | 11.8 |  |
| Ridderstrale[80] | 2014 | Glimeperide | 780 | 56 | N.R. | 24 |
|  |  | Empagliflozin | 769 | 56 | N.R. |  |
| Ristic[81] | 2007 | Gliclazide | 118 | 61 | 7.5 | 12 |
|  |  | Nateglinide | 129 | 61 | 7.6 |  |
| Rosenstock[82] | 2013 | Glipizide | 219 | 69 | 7.4 | 12 |
|  |  | Alogliptin | 222 | 69 | 7.5 |  |
| Tolman[83] | 2009 | Glibenclamide | 1057 | 55 | 9.5 | 36 |
|  |  | Pioglitazone | 1063 | 55 | 9.5 |  |
| UKPDS[2] | 1998 | Glibenclamide | 615 | 54 | 6.3 | 133 |
|  |  | Diet | 911 | 54 | 6.2 |  |
|  |  | Insulin | 896 | 54 | 6.1 |  |
| Vahatalo[84] | 2007 | Glipizide | 15 | 62 | 9.6 | 12 |
|  |  | Metformin | 26 | 62 | 9.8 |  |
|  |  | Nothing (all arms on insulin) | 11 | 62 | 10.0 |  |
