# Supplementary material for: The Association between Sulfonylurea Use and All-Cause and Cardiovascular Mortality: A Meta-Analysis with Trial Sequential Analysis of Randomized Clinical Trials
Source: PLoS Med. 2016 Apr 12;13(4):e1001992. doi: 10.1371/journal.pmed.1001992 (PMC4829174; doi:10.1371/journal.pmed.1001992)
Supplement: S1 Text — (PDF) [file pmed.1001992.s008.pdf]

PROSPERO International prospective register of systematic reviews

Review title and timescale

1 Review title
Give the working title of the review. This must be in English. Ideally it should state succinctly the interventions or exposures being reviewed and the associated health or social problem being addressed in the review.
Sulphonylurea in type 2 diabetes and all-cause mortality

2 Original language title
For reviews in languages other than English, this field should be used to enter the title in the language of the review. This will be displayed together with the English language title.

3 Anticipated or actual start date
Give the date when the systematic review commenced, or is expected to commence.
01/03/2014

4 Anticipated completion date
Give the date by which the review is expected to be completed.
01/03/2015

5 Stage of review at time of this submission
Indicate the stage of progress of the review by ticking the relevant boxes. Reviews that have progressed beyond the point of completing data extraction at the time of initial registration are not eligible for inclusion in PROSPERO. This field should be updated when any amendments are made to a published record.

The review has not yet started ×

Review stage	Started	Completed
Preliminary searches	Yes	Yes
Piloting of the study selection process	Yes	Yes
Formal screening of search results against eligibility criteria	Yes	No
Data extraction	No	No
Risk of bias (quality) assessment	No	No
Data analysis	No	No

Provide any other relevant information about the stage of the review here.

Review team details

6 Named contact
The named contact acts as the guarantor for the accuracy of the information presented in the register record.
Mr Rados

7 Named contact email
Enter the electronic mail address of the named contact.
dvarvaki@gmail.com

8 Named contact address
Enter the full postal address for the named contact.
Ramiro Barcelos, 2350; Serviço de Endocrinologia do Hospital de Clínicas de Proto Alegre

9 Named contact phone number
Enter the telephone number for the named contact, including international dialing code.
555133325188

10 Organisational affiliation of the review
Full title of the organisational affiliations for this review, and website address if available. This field may be completed as 'None' if the review is not affiliated to any organisation.
Serviço de Endocrinologia do HCPA

Website address:
http://www.diabetesendocrinologia.org.br

11 Review team members and their organisational affiliations
Give the title, first name and last name of all members of the team working directly on the review. Give the organisational affiliations of each member of the review team.

Title	First name	Last name	Affiliation
Mrs	Dimitris	Rados	UFRGS
Professor	Cristiane	Leitão	UFRGS/HCPA
Miss	Lana	Pinto	UFRGS
Professor	Luis Henrique	Canani	UFRGS/HCPA
Professor	Jorge Luis	Gross	UFRGS/HCPA

12 Funding sources/sponsors
Give details of the individuals, organizations, groups or other legal entities who take responsibility for initiating, managing, sponsoring and/or financing the review. Any unique identification numbers assigned to the review by the individuals or bodies listed should be included.
FIPE-HCPA

13 Conflicts of interest
List any conditions that could lead to actual or perceived undue influence on judgements concerning the main topic investigated in the review.
Are there any actual or potential conflicts of interest?
Yes

J.L.G. has received grants or research support from Eli Lilly, Bristol-Myers Squibb, Boehringer Ingelheim, GlaxoSmithKline, Novo Nordisk, and Janssen, and has served as a board member for Boehringer Ingelheim, Eli Lilly, and Novo Nordisk. LHC has served on a board for Janssen Cilag; RR has served as a consultant for Merck and Novo Nordisk, received grants from Merck and Novo Nordisk, and received payment for lectures for Eli Lilly

14 Collaborators
Give the name, affiliation and role of any individuals or organisations who are working on the review but who are not listed as review team members.

Title	First name	Last name	Organisation details

Review methods

15 Review question(s)
State the question(s) to be addressed / review objectives. Please complete a separate box for each question.
Are the sulphonylureas associated with increased mortality in the treatment of patients with type 2 diabetes?

16 Searches
Give details of the sources to be searched, and any restrictions (e.g. language or publication period). The full search strategy is not required, but may be supplied as a link or attachment.
PubMed EMBASE The Cochrane database ADA and EASD abstracts

17 URL to search strategy
If you have one, give the link to your search strategy here. Alternatively you can e-mail this to PROSPERO and we will store and link to it.

I give permission for this file to be made publicly available
No

18 Condition or domain being studied
Give a short description of the disease, condition or healthcare domain being studied. This could include health and wellbeing outcomes.
Type 2 diabetes

19 Participants/population

Give summary criteria for the participants or populations being studied by the review. The preferred format includes details of both inclusion and exclusion criteria.

Patients with type 2 diabetes

20 Intervention(s), exposure(s)

Give full and clear descriptions of the nature of the interventions or the exposures to be reviewed

Sulphonylurea

21 Comparator(s)/control

Where relevant, give details of the alternatives against which the main subject/topic of the review will be compared (e.g. another intervention or a non-exposed control group).

Other treatment options (biguanides, insulin, DPP-4 inhibtors, GLP-1 analogs, SGLT2 inhibitor, placebo)

22 Types of study to be included initially

Give details of the study designs to be included in the review. If there are no restrictions on the types of study design eligible for inclusion, this should be stated.

Randomized clinical trials

23 Context

Give summary details of the setting and other relevant characteristics which help define the inclusion or exclusion criteria.

A systematic review of randomized clinical trials of sulphonylureas in the treatment of type 2 diabetes

24 Primary outcome(s)

Give the most important outcomes.

Total and cardiovascular mortality

Give information on timing and effect measures, as appropriate.

25 Secondary outcomes

List any additional outcomes that will be addressed. If there are no secondary outcomes enter None.

Cardiovascular events, severe hypoglycaemia, hospitalization, glycemic control

Give information on timing and effect measures, as appropriate.

26 Data extraction, (selection and coding)

Give the procedure for selecting studies for the review and extracting data, including the number of researchers involved and how discrepancies will be resolved. List the data to be extracted.

Two researchers (D.R. and L.P.) will review the records independently searching for clinical trials evaluating the effects of sulfonylureas in type 2 diabetic patients. After individual reviewing, the selection of studies will be compared, the Kappa statistic will be calculated and discrepancies will be resolved be agreement or by a third researcher (C.B.L.)

27 Risk of bias (quality) assessment

State whether and how risk of bias will be assessed, how the quality of individual studies will be assessed, and whether and how this will influence the planned synthesis.

The selected studies will be evaluated according to the Cochrane Collaborations tool and GRADE system. We are planning sub-analysis according to the quality (main results including and excluding low quality trials).

28 Strategy for data synthesis

Give the planned general approach to be used, for example whether the data to be used will be aggregate or at the level of individual participants, and whether a quantitative or narrative (descriptive) synthesis is planned. Where appropriate a brief outline of analytic approach should be given.

We are planning to aggregated data at the study level. Also a trial sequential analysis is planned.

29 Analysis of subgroups or subsets

Give any planned exploration of subgroups or subsets within the review. 'None planned' is a valid response if no subgroup analyses are planned.

Short x long duration trials High quality x low quality trials Each sulfonylurea individually Sulfonylurea as first drug

Sulfonylurea as adjunct treatment with metformin x metformin + other treatments

Review general information

30 Type of review
Select the type of review from the drop down list.
Intervention

31 Language
Select the language(s) in which the review is being written and will be made available, from the drop down list. Use the control key to select more than one language.
English

Will a summary/abstract be made available in English?
Yes

32 Country
Select the country in which the review is being carried out from the drop down list. For multi-national collaborations select all the countries involved. Use the control key to select more than one country.
Brazil

33 Other registration details
Give the name of any organisation where the systematic review title or protocol is registered together with any unique identification number assigned. If extracted data will be stored and made available through a repository such as the Systematic Review Data Repository (SRDR), details and a link should be included here.

34 Reference and/or URL for published protocol
Give the citation for the published protocol, if there is one.
Give the link to the published protocol, if there is one. This may be to an external site or to a protocol deposited with CRD in pdf format.

I give permission for this file to be made publicly available
No

35 Dissemination plans
Give brief details of plans for communicating essential messages from the review to the appropriate audiences.
We plan on publishing our results in a medical journal and presenting them in congress and/or meetings

Do you intend to publish the review on completion?
Yes

36 Keywords
Give words or phrases that best describe the review. (One word per box, create a new box for each term)
sulphonylurea

type 2 diabetes

mortality

cardiovascular events

37 Details of any existing review of the same topic by the same authors
Give details of earlier versions of the systematic review if an update of an existing review is being registered, including full bibliographic reference if possible.
There is a previous systematic review and meta-analysis by another group showing greater risk for cardiovascular mortality when comparing first generation with placebo. However, this analysis has some limitations in that it evaluated sulfonyureas only as monotherapy, and did not use a network meta-analysis technique.

38 Current review status

Review status should be updated when the review is completed and when it is published.
Ongoing

39 Any additional information
Provide any further information the review team consider relevant to the registration of the review.

40 Details of final report/publication(s)
This field should be left empty until details of the completed review are available.
Give the full citation for the final report or publication of the systematic review.
Give the URL where available.
