# Supplementary material for: The Association between Sulfonylurea Use and All-Cause and Cardiovascular Mortality: A Meta-Analysis with Trial Sequential Analysis of Randomized Clinical Trials
Source: PLoS Med. 2016 Apr 12;13(4):e1001992. doi: 10.1371/journal.pmed.1001992 (PMC4829174; doi:10.1371/journal.pmed.1001992)
Supplement: S2 Text — (DOC) [file pmed.1001992.s009.doc]

| **Section/topic** | **#** | **Checklist item** | **Reported on page #** |
| --- | --- | --- | --- |
| **TITLE** | | |  |
| Title | 1 | Identify the report as a systematic review, meta-analysis, or both.  “*The association between sulfonylurea use and all-cause and cardiovascular mortality: a meta-analysis with trial sequential analysis of randomized clinical trials”* | §1 |
| **ABSTRACT** | | |  |
| Structured summary | 2 | Provide a structured summary including, as applicable: background; objectives; data sources; study eligibility criteria, participants, and interventions; study appraisal and synthesis methods; results; limitations; conclusions and implications of key findings; systematic review registration number.  “*BACKGROUND: Sulfonylureas are an effective and inexpensive treatment for type 2 diabetes. There is conflicting data about the safety of these drugs regarding mortality and cardiovascular outcomes. The objective of the present study was to evaluate the safety of sulfonylureas most frequently used, and to analyse if the available sample is powered enough to support the results through trial sequential analysis (TSA).*  *METHODS AND FINDINGS: Electronic databases were reviewed from 1946 (EMBASE) or 1966 (MEDLINE) up to 31st December 2014. Randomised clinical trials (RCT) of at least 52 weeks in duration evaluating second- or third-generation sulfonylureas in the treatment of adults with type 2 diabetes and reporting outcomes of interest were included. Primary outcomes were all-cause and cardiovascular mortality. Additionally, myocardial infarction and stroke events were evaluated. Data were summarized with Peto odds ratio and the reliability of the results was evaluated with TSA. Forty-seven RCTs with 37,650 patients and 890 deaths in total were included. Sulfonylureas were not associated with all-cause (OR 1.12 [95% CI 0.96 to 1.30]) or cardiovascular mortality (OR 1.12 [95% CI 0.87 to 1.42]). Sulfonylureas were also not associated with increased risk of myocardial infarction (OR 0.92 [95% CI 0.76 to 1.12]) or stroke (OR 1.16 [95% CI 0.81 to 1.66]). TSA could discard an absolute difference of 0.5% between the treatments, which was considered the minimal clinically significant difference. The major limitation of this review was the inclusion of studies not designed to evaluate safety outcomes.*  *CONCLUSION: Sulfonylureas are not associated with increased risk for all-cause and cardiovascular mortality, myocardial infarction or stroke. Current evidence supports the safety of sulfonylureas; an absolute risk of 0.5% could be firmly discarded. PROSPERO registry CRD42014004330.* ” | §6-8 |
| **INTRODUCTION** | | |  |
| Rationale | 3 | Describe the rationale for the review in the context of what is already known.  “*There are concerns regarding the safety of sulfonylureas that have persisted since the results of the first randomized controlled trial (RCT) in the evaluation of diabetes treatment (University Group Diabetes Program)[4] until the present time [5-7].*”  “*Recent meta-analyses evaluating the safety of sulfonylureas as group [18-21] or in association with metformin [22] also reported contradictory results. Probably, this was due to the inclusion of observational studies [21,22], inclusion of first generation sulfonylureas [19,20] and lack of evaluation of the risk of type II error [18,20,21]. Studies that included second or third generation sulfonylureas did not reported higher risk [18-21].*”  “*Trial sequential analysis (TSA) is a tool that is increasingly being used [23] to assess whether optimal sample sizes − and benefit or harm boundaries − have been reached by an available sample assuming a minimal clinical significant difference [24]. It has the potential to increase data reliability [24], and its use might be of great benefit in determining whether the currently evaluable evidence about the safety of sulfonylureas is enough to discard falsely positive or negative conclusions [25].*” | §11, 13 and 14 |
| Objectives | 4 | Provide an explicit statement of questions being addressed with reference to participants, interventions, comparisons, outcomes, and study design (PICOS).  “*the aim of this study was to evaluate the safety of second and third generation sulfonylureas use in all-cause and cardiovascular mortality and cardiovascular events (myocardial infarction and stroke), and to quantify the statistical reliability of available data*”  “*The present study was intended to evaluate the overall safety of most frequently used sulfonylureas (both second- and third-generation) in type 2 diabetes through a review of RCTs. Therefore, the search strategy included the terms ‘type 2 diabetes’, ‘sulfonylureas’ (second- and third-generation) and used the recommended, highly sensitive Cochrane Collaboration strategy for RCT systematic reviews*” | §15 and 21 |
| **METHODS** | | |  |
| Protocol and registration | 5 | Indicate if a review protocol exists, if and where it can be accessed (e.g., Web address), and, if available, provide registration information including registration number.  “*(…) registered it on the PROSPERO registry (CRD42014004330).*” | §18 |
| Eligibility criteria | 6 | Specify study characteristics (e.g., PICOS, length of follow-up) and report characteristics (e.g., years considered, language, publication status) used as criteria for eligibility, giving rationale.  “*We included RCTs that evaluated patients with type 2 diabetes who were randomized to receive a second- or third-generation sulfonylurea for at least 52 weeks, and which reported all-cause or cardiovascular mortality, myocardial infarction or stroke data.*”  “*We searched the on-line databases of MEDLINE (through PubMed), EMBASE, and the Cochrane Library from inception up to December 2014*” | §21 and 22 |
| Information sources | 7 | Describe all information sources (e.g., databases with dates of coverage, contact with study authors to identify additional studies) in the search and date last searched.  “*We searched the on-line databases of MEDLINE (through PubMed), EMBASE, and the Cochrane Library from inception up to December 2014, as well as a manual review of reference lists of published studies. (…) We also searched the clinicaltrials.org registry and the 2014 abstract books of international diabetes meetings (American Diabetes Association [ADA] and European Association for the Study of Diabetes [EASD]) for unpublished studies. No time period restrictions were made.*” | §22 |
| Search | 8 | Present full electronic search strategy for at least one database, including any limits used, such that it could be repeated.  “*("Glyburide"[Mesh]) OR ("glibornuride" [Supplementary Concept]) OR ("Glipizide"[Mesh]) OR ("gliquidone" [Supplementary Concept]) OR ("glisoxepide" [Supplementary Concept]) OR ("glyclopyramide" [Supplementary Concept]) OR ("glimepiride" [Supplementary Concept]) OR ("Gliclazide"[Mesh]) AND ("Diabetes Mellitus, Type 2"[Mesh]) AND (randomized controlled trial[pt] OR controlled clinical trial[pt] OR randomized controlled trials[mh] OR random allocation[mh] OR double-blind method[mh] OR single-blind method[mh] OR clinical trial[pt] OR clinical trials[mh] OR (“clinical trial”[tw]) OR ((singl*[tw] OR doubl*[tw] OR trebl*[tw] OR tripl*[tw]) AND (mask*[tw] OR blind*[tw])) OR (“latin square”[tw]) OR placebos[mh] OR placebo*[tw] OR random*[tw] OR research design[mh: noexp] OR follow-up studies[mh] OR prospective studies[mh] OR cross-over studies[mh] OR control*[tw] OR prospectiv*[tw] OR volunteer*[tw]) NOT (animal[mh] NOT human[mh])*” | Appendix, S1 Table |
| Study selection | 9 | State the process for selecting studies (i.e., screening, eligibility, included in systematic review, and, if applicable, included in the meta-analysis).  “*Two investigators (D.V.R. and L.C.P.) independently evaluated the titles and abstracts of the articles retrieved using the search process. Abstracts that did not meet the inclusion criteria or meeting exclusion criteria were discarded. We selected the remaining studies for full text evaluation and data extraction. Any disagreements regarding inclusion or exclusion of a study were solved by consensus and, if doubt persisted, a third reviewer (C.B.L) evaluated the reference.*” | §27 |
| Data collection process | 10 | Describe method of data extraction from reports (e.g., piloted forms, independently, in duplicate) and any processes for obtaining and confirming data from investigators.  “*We used a standardized form to extract the following details from retrieved studies*” | §28 |
| Data items | 11 | List and define all variables for which data were sought (e.g., PICOS, funding sources) and any assumptions and simplifications made.  “*(…) first author’s name, publication year and journal, study characteristics (i.e. comparator, co-intervention), patient characteristics (mean age, proportion of men/women, proportion of patients with hypertension, dyslipidaemia and active smoking), study methodology (intervention dosages, frequency and duration), number of patients included and lost to follow-up, and number of patients with outcomes of interest (all-cause and cardiovascular death, myocardial infarction and stroke).*” | §28 |
| Risk of bias in individual studies | 12 | Describe methods used for assessing risk of bias of individual studies (including specification of whether this was done at the study or outcome level), and how this information is to be used in any data synthesis.  “*We assessed the included studies in six domains according to The Cochrane Collaboration’s tool for assessing risk of bias [26,28]: i) random sequence generation, ii) allocation concealment, iii) blinding, iv) incomplete outcome data, v) selective reporting and vi) other bias; for other bias we evaluated if the study was conducted with funding support from the pharmaceutical industry. We evaluated the quality of the evidence for each meta-analysis using the Grading of Recommendations, Assessment, Development and Evaluations (GRADE) approach. The quality of evidence was classified as ‘high’, ‘moderate’, ‘low’ or ‘very low’.*” | §30 |
| Summary measures | 13 | State the principal summary measures (e.g., risk ratio, difference in means).  “*(…) the studies were summarized using the Peto OR method.*” | §36 |
| Synthesis of results | 14 | Describe the methods of handling data and combining results of studies, if done, including measures of consistency (e.g., I^2^) for each meta-analysis.  “*We compared the outcomes of interest in patients treated with sulfonylureas with a control group (diet, placebo or other antihyperglycemic medication).*”  “*(…) the studies were summarized using the Peto OR method. This method seems to be better suited to these situations, especially when the incidence of events is near 1% and the effects of intervention are of a small magnitude [33].*”  “*We evaluated the heterogeneity using a Cochran Q test with a threshold P-value of 0.1 and an I2 test, with a value >50% indicating of high heterogeneity.*”  “*To evaluate if the present meta-analysis had sufficient sample size for establishing firm conclusions about the effect of interventions [24,25], we performed TSA for the major outcomes.*” | §33  §36  §38  §35 |

Page 1 of 2

| **Section/topic** | **#** | **Checklist item** | **Reported on page #** |
| --- | --- | --- | --- |
| Risk of bias across studies | 15 | Specify any assessment of risk of bias that may affect the cumulative evidence (e.g., publication bias, selective reporting within studies).  “*We assessed small study bias by using a contour-enhanced funnel plot and asymmetry by using Begg and Egger tests. A significant bias was considered if the P<0.10. A trim-and-fill computation was used to estimate the effect of missing studies on the interpretation of results.*” | §39 |
| Additional analyses | 16 | Describe methods of additional analyses (e.g., sensitivity or subgroup analyses, meta-regression), if done, indicating which were pre-specified.  “*As a sensibility analysis we performed the analysis with Mantel-Haenszel*”  “*We also performed a meta-analysis separating the controls in classes (diet or placebo and active comparators). We also accessed the use of sulfonylureas as first-line treatment (monotherapy), second-line treatment (in addition to some medication) or other treatment (when the study did not defined the line of treatment as inclusion criteria). Furthermore, as sulfonylureas are commonly used as a second agent in addition to metformin [1,33,34], we assessed the effects of sulfonylureas when used as an add-on to metformin. We also did exploratory meta-analyses for each sulfonylurea (glibenclamide, glimepiride, glipizide and gliclazide).*”  “*(…)it is suggested that a sensitivity analysis with continuity correction is performed [37]. However, TSA software does include double-zero events trials in the analysis, using empirical continuity correction. This is performed adding a constant in the number of events and non-events in both treatment arms. This constant is calculated for each trial and each arm and this calculation is based on the OR of the meta-analysis (without the zero events studies to be corrected) and the randomization ratio of the study that needs the empirical continuity correction [25]. Therefore, although our forest plots were constructed using the Peto OR analysis (double-zero studies not plotted) double-zero studies were included in the TSA analysis and graphics.*” | §36  §33  §37 |
| **RESULTS** | | |  |
| Study selection | 17 | Give numbers of studies screened, assessed for eligibility, and included in the review, with reasons for exclusions at each stage, ideally with a flow diagram.  “*We identified 5572 studies through both the literature and manual searches (Fig 1). After excluding duplicate references and reviewing titles and abstracts, we selected 192 references for full-text evaluation. One-hundred-and-nine trials either did not meet the inclusion criteria or met the exclusion criteria. The main reasons for exclusions were: short duration (40 references, 37%), duplicated records (24 references, 22%) and non-randomised study (17 references, 15%). In addition, 36 studies did not report outcome data and this data was not forthcoming after contacting the corresponding authors. We contacted 19 authors from 28 studies. Five authors answered our request, but none of them provided additional data. These studies represented only 10% of the total sample. The reviewers had a high agreement rate (κ=0.917).*” | §43  Figure 1 |
| Study characteristics | 18 | For each study, present characteristics for which data were extracted (e.g., study size, PICOS, follow-up period) and provide the citations.  S2 Table | S2 Table |
| Risk of bias within studies | 19 | Present data on risk of bias of each study and, if available, any outcome level assessment (see item 12).  S2 Figure | S2 Figure |
| Results of individual studies | 20 | For all outcomes considered (benefits or harms), present, for each study: (a) simple summary data for each intervention group (b) effect estimates and confidence intervals, ideally with a forest plot.  Fig 2-5 and S3-5 Fig | Fig 2-5 and S3-5 Fig |
| Synthesis of results | 21 | Present results of each meta-analysis done, including confidence intervals and measures of consistency.  “*Our meta-analysis did not show a significant association between use of sulfonylureas and all-cause (OR 1.12 [95% CI 0.96 to 1.30]) (Fig 2) or cardiovascular mortality (OR 1.12 [95% CI 0.87 to 1.42]) (S3 Fig). Both analyses have low heterogeneity (all-cause mortality: I2 = 0% [95% CI 0% to 17%], P for heterogeneity = 0.67; cardiovascular mortality: I2 = 12% [95% CI 0% to 20%], P for heterogeneity = 0.30). The inclusion of double-zero studies with empirical continuity correction analysis did not affect the results (OR 1.11 [95% CI 0.96 to 1.29] and OR 1.12 [95% CI 0.87 to 1.42] for all-cause and cardiovascular mortality, respectively).*”  “*A smaller number of trials reported myocardial infarction and stroke data (23 studies each, comprising 26,521 and 26,175 patients for myocardial infarction and stroke, respectively). We found no significant difference for myocardial infarction in patients treated with sulfonylureas (OR 0.92 [95% CI 0.76 to 1.12]). (…) In addition, no significant association was observed between sulfonylureas and stroke (OR 1.16 [95% CI 0.81 to 1.66]).*” | §49  §53  Fig 2 and S3 Fig |
| Risk of bias across studies | 22 | Present results of any assessment of risk of bias across studies (see Item 15).  “*We identified small study bias for all-cause mortality. Despite this, the results were unaffected by the trim-and-fill computation: in reality, the point estimation after the computation of theoretical unpublished studies for all-cause mortality was smaller (OR 1.08 [95% CI 0.93 to 1.25]). There was no small study bias for cardiovascular mortality.*”  “*Small study bias was present for myocardial infarction, but the results were similar with the trim-and-fill computation (OR 0.90 [95% CI 0.74 to 1.09]). No small study bias was identified for stroke events.* “ | §51  §53 |
| Additional analysis | 23 | Give results of additional analyses, if done (e.g., sensitivity or subgroup analyses, meta-regression [see Item 16]).  “*The inclusion of double-zero studies with empirical continuity correction analysis did not affect the results (OR 1.11 [95% CI 0.96 to 1.29] and OR 1.12 [95% CI 0.87 to 1.42] for all-cause and cardiovascular mortality, respectively).*”  “*We intended to evaluate long-term safety of sulfonylureas, so to address if longer studies might show different results, we restricted the analysis for studies with follow-up of at least 2 years. The results were similar for all-cause (OR 1.05 [95% CI 0.89 to 1.24]) and cardiovascular mortality (OR 1.07 [95% CI 0.83 to 1.39]).*”  “*Including double-zero studies with empirical continuity correction left the results unaffected (OR 0.92 [95% CI 0.76 to 1.12). (…)The inclusion of double-zero studies with empirical continuity correction did not change these results as well (OR 1.16 [95% CI 0.89 to 1.63]).*“  “*Sulfonylureas as add-on to metformin were considered safe in terms of overall and cardiovascular mortality (Fig 5) with little heterogeneity: OR 1.26 (95% CI 0.94 to 1.68; I2 = 0% [95% CI 0% to 31%], P for heterogeneity = 0.97) for all-cause mortality and OR 1.40 (95% CI 0.61 to 3.22; I2 = 6% [95% CI 0% to 52%], P for heterogeneity = 0.38) for cardiovascular mortality. Including double-zero studies with empirical continuity correction in the analysis did not change these results. All studies in these analyses had active comparators against sulfonylureas.*”  “*As an exploratory evaluation, all-cause mortality analysis for each individual sulfonylurea is shown in supporting information (S5 Fig). Results are similar for cardiovascular mortality. In both analyses, heterogeneity was small. Glipizide was associated with increased all-cause (OR 1.68 [95% CI 1.06 to 2.66]) and cardiovascular mortality (OR 2.1 [95% CI 1.09 to 3.72]), but these analyses are based in a small number of patients and studies. A sensitivity analysis excluding glipizide trials from the main analyses was performed. We observed a reduction in ORs for all-cause (OR 1.03 [95% CI 0.86 to 1.23] and cardiovascular mortality (OR 1.00 [95% CI 0.77 to 1.30]). Of note, the futility boundary was still reached in this situation.*“  “*TSA evaluates if there is enough information size to establish firm conclusions and this analysis was performed for the main outcomes in this review. For all-cause and cardiovascular mortality TSA showed that a NNH of 200 could be discarded, as the number of patients evaluated for all-cause (n = 37,650) and cardiovascular mortality (n = 21,893) surpassed the optimal sample sizes (n = 29,819 for all-cause mortality and n = 21,593 for cardiovascular mortality) (Fig 6A and 6B). The combination of sulfonylureas and metformin was evaluated with TSA as well. The Z-curve surpassed the optimal sample size boundary and a NNH of 200 could be discarded for all-cause mortality (Fig 6C) but not for cardiovascular mortality. Similarly, for myocardial infarction and stroke the futility boundaries were reached..*” | §49  §51  §53  §61 and Fig 5  §64 and S5 Fig  §67 and Fig 6 |
| **DISCUSSION** | | |  |
| Summary of evidence | 24 | Summarize the main findings including the strength of evidence for each main outcome; consider their relevance to key groups (e.g., healthcare providers, users, and policy makers).  “*The GRADE quality of evidence for all-cause and cardiovascular mortality was high. The identified publication bias does not appear to have skewed the results of the meta-analysis. Financial support from pharmaceutical industry is a conservative bias, as it might have increased the risk of benefit for the comparator drug [81].We graded the myocardial infarction and stroke meta-analysis as being of moderate quality. As these outcomes are at greater risk of being skewed due to the identified bias (especially due to underreporting and misdiagnosis) we downgraded the evidence by one point.* “  “*The data presented here suggest that most frequently used sulfonylureas (second and third generations) are not associated with increased all-cause and cardiovascular mortality in patients with type 2 diabetes. By using TSA we were able to discard harm at a rate of 1 in every 200 treated patients (i.e. 0.5% of absolute risk) for mortality (all-cause and cardiovascular) and major events (myocardial infarction and stroke). We defined this as the minimal clinically significant difference based on previous study [86]. Furthermore, this finding did not change when sulfonylureas were compared with almost every drug class currently available for the treatment of type 2 diabetes or as an add-on to metformin.*” | §71 and 72  §74 |
| Limitations | 25 | Discuss limitations at study and outcome level (e.g., risk of bias), and at review-level (e.g., incomplete retrieval of identified research, reporting bias).  “*Some limitations of the present study must be acknowledged. Unfortunately, we were not able to include all the identified studies in the meta-analyses because the mortality outcomes were not available, even after trying to contact the authors. However, these studies represented only 10% of the study population. It seems unlikely that these data would change the results as optimal sample size was reached for most analyses. We also could not include 3 studies due to language restrictions. Some of our analyses were explorative ones (individual sulfonylureas, individual comparators) and the results should be interpreted and used in the clinical practice with caution. To access if eligible studies were published in the last year, we updated the review of databases (MEDLINE, EMBASE and Cochrane Library) with the original strategy until 9th February 2016. We identified one new study that would fulfill the inclusion criteria of this review [88]. This study included 720 patients randomized to glimepiride or saxagliptin. There was only one death in each group; hence this additional data did not change our result. Finally, most studies were not designed for accessing long-term safety endpoints but all of them have a duration of 52 weeks or more, which partially controls for this limitation. Although 52 weeks may be a short time frame to identify mortality and cardiovascular outcomes, the analysis with longer studies (at least 2 years) did not change the results. Finally, as the lower limit of most results is lower than 1, but close to it, different analysis methods may lead to different results. However, we decreased the uncertainty by performing sensitivity analyses and also explored the consistency of the results by using TSA.*” | §78 |
| Conclusions | 26 | Provide a general interpretation of the results in the context of other evidence, and implications for future research.  “*Other systematic reviews also evaluated this topic [18-22]. Although some of these studies identified increased risk of occurrence of mortality or cardiovascular events with sulfonylurea use [19,20,22], other did not found an increased risk [18,21]. These contradictory results may be explained by the inclusion of first generation sulfonylureas [19,20], observational studies [21,22] and short-term studies [18-21]. Furthermore, most systematic reviews did not evaluate if the data presented had enough power to support the conclusions [18,20,21]. We included only RCTs evaluating sulfonylureas from second and third generations as monotherapy or in combination. We chose to include only these sulfonylureas, because they are more frequently used than the first generation [8]; alone or in combination with metformin [10].*”  “Another important unresolved question is which drug should be added to patients who are failing metformin monotherapy. To date, no antihyperglycemic agent reduced mortality or cardiovascular events in association with metformin. Even the recent published trials of dipeptidyl peptidase-4 inhibitors in patients with type 2 diabetes and high cardiovascular risk did not reduce cardiovascular events [12,13,90], but there was a concern regarding heart failure incidence in two of them [12,91]. Our data show that second and third generations sulfonylureas are a safe option, but we hope that newer drugs will do better than that and will be able to decrease cardiovascular events and mortality risks when compared to sulfonylureas. Although EMPA-REG study did not directly explored this issue, the results suggest that empagliflozin might be this drug, as it was able to reduce the risk of cardiovascular events and mortality (all cause and cardiovascular)[92]. To clarify the question of which should be the preferred drug for patients failing metformin, The Cardiovascular Outcome Study of Linagliptin Versus Glimepiride in Patients With Type 2 Diabetes (CAROLINA) and the Glycemia Reduction Approaches in Diabetes: A Comparative Effectiveness Study (GRADE) results are awaited [93,94] as they will further evaluate sulfonylurea effect against newer drug classes in the long term.” | §75  §80 |
| **FUNDING** | | |  |
| Funding | 27 | Describe sources of funding for the systematic review and other support (e.g., supply of data); role of funders for the systematic review.  “*This study was funded by the Conselho Nacional de Desenvolvimento Científico e Tecnológico (CNPq). Support for the publication fee was provided. CNPq had no role in the design and conduct of the study; the extraction, management, analysis, or interpretation of the data; or the preparation, review, or approval of the manuscript.*” | Funding information section; not shown in main text. |

*From:*  Moher D, Liberati A, Tetzlaff J, Altman DG, The PRISMA Group (2009). Preferred Reporting Items for Systematic Reviews and Meta-Analyses: The PRISMA Statement. PLoS Med 6(6): e1000097. doi:10.1371/journal.pmed1000097

For more information, visit: **www.prisma-statement.org**.

Page 2 of 2
